# Disorders of phospholipid metabolism: an emerging class of mitochondrial disease due to defects in nuclear genes

**DOI:** 10.3389/fgene.2015.00003

**Published:** 2015-02-03

**Authors:** Ya-Wen Lu, Steven M. Claypool

**Affiliations:** Department of Physiology, School of Medicine, Johns Hopkins UniversityBaltimore, MD, USA

**Keywords:** mitochondrial disease, phospholipid metabolism, cardiolipin, Barth syndrome, MEGDEL, DCMA, Sengers syndrome, hereditary spastic paraplegia

## Abstract

The human nuclear and mitochondrial genomes co-exist within each cell. While the mitochondrial genome encodes for a limited number of proteins, transfer RNAs, and ribosomal RNAs, the vast majority of mitochondrial proteins are encoded in the nuclear genome. Of the multitude of mitochondrial disorders known to date, only a fifth are maternally inherited. The recent characterization of the mitochondrial proteome therefore serves as an important step toward delineating the nosology of a large spectrum of phenotypically heterogeneous diseases. Following the identification of the first nuclear gene defect to underlie a mitochondrial disorder, a plenitude of genetic variants that provoke mitochondrial pathophysiology have been molecularly elucidated and classified into six categories that impact: (1) oxidative phosphorylation (subunits and assembly factors); (2) mitochondrial DNA maintenance and expression; (3) mitochondrial protein import and assembly; (4) mitochondrial quality control (chaperones and proteases); (5) iron–sulfur cluster homeostasis; and (6) mitochondrial dynamics (fission and fusion). Here, we propose that an additional class of genetic variant be included in the classification schema to acknowledge the role of genetic defects in phospholipid biosynthesis, remodeling, and metabolism in mitochondrial pathophysiology. This seventh class includes a small but notable group of nuclear-encoded proteins whose dysfunction impacts normal mitochondrial phospholipid metabolism. The resulting human disorders present with a diverse array of pathologic consequences that reflect the variety of functions that phospholipids have in mitochondria and highlight the important role of proper membrane homeostasis in mitochondrial biology.

## MITOCHONDRIA AND DISEASE

The mitochondrion is the primary generator of adenosine triphosphate (ATP) in eukaryotes. In addition to oxidative phosphorylation (OXPHOS), the mitochondrion is involved in a wide range of essential cellular processes. The organelle is the home for the tricarboxylic acid cycle, fatty acid beta-oxidation, iron–sulfur cluster biogenesis, a portion of the urea cycle, and steps in the porphyrin and pyrimidine biosynthetic pathways. Moreover, mitochondria have important roles in Ca^2+^ buffering and thus, Ca^2+^ signaling, are a major producer (a consequence of OXPHOS) and scavenger of reactive oxygen species (ROS), and are intimately involved in programmed cell death (Kroemer et al., 1995; Green and Kroemer, 2004). Finally, the mitochondrion directly contributes to cellular phospholipid metabolism by hosting machinery that can produce at least four distinct phospholipids: phosphatidic acid (PA), phosphatidylglycerol (PG), cardiolipin (CL), and phosphatidylethanolamine (PE). Given the myriad of roles the mitochondrion plays in maintaining cellular homeostasis, it is no surprise that defects in mitochondrial function lead to a broad spectrum of diseases (Wong, 2007).

Mitochondrial disorders, first recognized in Luft et al. (1962), are the most common source of inborn errors of metabolism (Naviaux, 2004; Schaefer et al., 2008; Foundation, 2014). Primary mitochondrial disorders can be caused by mutation of genes encoded by either mitochondrial (mt)DNA or nuclear (n)DNA (but whose protein product resides in the mitochondrion). Thus, while the discovery of pathogenic mtDNA mutations in the 1980s greatly facilitated understanding of maternally-inherited OXPHOS disease (Holt et al., 1988; Wallace et al., 1988), the clinical and genetic heterogeneity of mitochondrial disease still complicates efforts to diagnose, manage, and treat affected patients (Wong, 2007; Calvo and Mootha, 2010; DaRe et al., 2013).

Human mtDNA encodes 13 OXPHOS subunits, 22 transfer RNAs, and two ribosomal RNAs (Anderson et al., 1981). In contrast, 99% of the mitochondrial proteome, which consists of over 1000 proteins (Mootha et al., 2003; Taylor et al., 2003; Zahedi et al., 2006; Johnson et al., 2007; Pagliarini et al., 2008; Calvo and Mootha, 2010; Calvo et al., 2012; Rhee et al., 2013; Hung et al., 2014), is encoded by the nuclear genome. Thus, not surprisingly, disorders that are caused by pathogenic mtDNA mutations [identified in 30 of the 37 mtDNA-encoded proteins (Ye et al., 2014b)] only comprise a fifth of the known mitochondrial diseases (Dimauro and Davidzon, 2005).

Mitochondrial dysfunction is not only defined by specific defects in mtDNA or in OXPHOS-associated processes. Mutations in enzymes involved in any number of the mitochondrion’s essential functions can lead to mitochondrial disease. Genetic variants that drive mitochondrial pathophysiology have been broadly classified into six categories: (1) OXPHOS-related (subunits and assembly factors; Ugalde et al., 2009); (2) mtDNA maintenance and expression (Jacobs and Turnbull, 2005); (3) mitochondrial biogenesis (Perez-Martinez et al., 2008); (4) mitochondrial quality control (chaperones and proteases; Koppen and Langer, 2007); (5) iron–sulfur cluster homeostasis (Lill et al., 2012); and (6) mitochondrial dynamics (fission and fusion; Chen and Chan, 2009). These categories are delineated by the pathways in which the impacted proteins partake but do not predict in any way known, the clinical presentation caused by any given genetic mutation.

Here, we propose that an additional class of genetic variants be included in the classification schema to acknowledge the role of defects in mitochondrial phospholipid metabolism as a cause of mitochondrial disease. This seventh class will encompass a small but notable group of Mendelian-inherited disorders that specifically impact normal mitochondrial phospholipid metabolism and thus highlight the important role of proper membrane homeostasis in mitochondrial physiology. PA, PG, CL, and PE have parts, if not all, of their biosynthetic pathways localized to the mitochondrion. Combined with the essential import of extra-mitochondrial phosphatidylcholine, phosphatidylserine and phosphatidylinositol, the mitochondrion requires and maintains a highly articulated lipid trafficking network. Therefore, it is no surprise that disruption of mitochondrial phospholipid metabolism can lead to mitochondrial dysfunction.

While the mitochondrion hosts a major PE biosynthetic pathway, to date, an inherited disease directly impinging on mitochondrial PE metabolism has not been demonstrated. As such, the biology of this important phospholipid will not be covered here. Instead, this review will only focus on those phospholipids which are impacted by mutations in genes encoded by the nuclear genome with defined or emerging roles in mitochondrial phospholipid metabolism.

## CARDIOLIPIN METABOLISM

### MITOCHONDRIAL MEMBRANES AND ASYMMETRY

Each mitochondrion has two highly specialized membranes, the outer mitochondrial membrane (OMM) and inner mitochondrial membrane (IMM), delineating two aqueous compartments, a dense internal matrix and an intermembrane space (IMS). Characteristic to mitochondrial membranes is a low phospholipid to protein ratio relative to other organelle membranes, high PC and PE content, cumulatively accounting for up to 80% of total lipid phosphorous, low amounts of sterols and sphingolipids, and enrichment of a unique phospholipid, CL (Horvath and Daum, 2013).

Commonly known as the signature phospholipid of mitochondria, CL is a phospholipid dimer that consists of a pair of PAs, each with two fatty acyl chains, bridged by a central glycerol moiety (Lecocq and Ballou, 1964; Schlame et al., 2000). The presence of four acyl chains per molecule provides the opportunity for an incredible diversity of CL molecular forms while the two phosphate groups confer upon the lipid a net –1 charge at physiological pH (Haines and Dencher, 2002). CL, like PE and PA, is a non-bilayer forming phospholipid; these lipids have much smaller hydrophilic head group diameters than their hydrophobic acyl chains, making them cone-shaped. Non-bilayer lipids participate in membrane fusion events (Verkleij et al., 1984), facilitate membrane bending (Gruner, 1985), and can impart order to surrounding lipids (Zeczycki et al., 2014). This distinguishes them from bilayer-forming lipids that have head groups and acyl chains that are of similar diameter.

Each mitochondrial membrane has a distinct protein population and phospholipid composition (Colbeau et al., 1971; Zinser et al., 1991; Zinser and Daum, 1995; Daum and Vance, 1997; Mootha et al., 2003; Taylor et al., 2003; Zahedi et al., 2006; Johnson et al., 2007; Pagliarini et al., 2008; Gebert et al., 2009; Rhee et al., 2013; Hung et al., 2014). In fact, the protein to phospholipid ratio differs significantly between the IMM and OMM, with the IMM being significantly more proteinaceous (Sperka-Gottlieb et al., 1988; Ardail et al., 1990; Simbeni et al., 1991). When purified, OMM and IMM vesicles have different shapes, structures, and lipid compositions (Hovius et al., 1990; Gebert et al., 2009). Notably, the OMM has relatively less CL than the IMM (Daum and Vance, 1997; de Kroon et al., 1997). Even within a membrane bilayer, there is asymmetry in the lipid composition between the two leaflets (Krebs et al., 1979; Harb et al., 1981; Cheneval et al., 1985; Sperka-Gottlieb et al., 1988; Hovius et al., 1993; Gallet et al., 1997). For instance, most of the associated PE and all of the low amounts of CL are on the cytoplasmic-facing side of OMM (Hovius et al., 1993).

The high protein to phospholipid ratio in the IMM reflects the sheer magnitude of essential processes that occur in its context. Embedded in the IMM is the OXPHOS system, three distinct translocation machineries (TIM22, TIM23, and OXA1), carrier proteins that mediate the flux of metabolites across the IMM, quality control proteases, and phospholipid metabolizing enzymes. In spite of this protein density, the IMM is an intact diffusion barrier that enforces the proton gradient generated by the electron transport chain. The stored power of the electrochemical gradient across the IMM is central to mitochondrial biology. Not only is it used as the source of energy for ATP production and harnessed to drive numerous transport processes, but in fact, the electrochemical gradient is required for mitochondrial biogenesis itself (Chacinska et al., 2009). Therefore, maintaining a proper lipid composition is likely not only required to support the functionality of the numerous proteins and protein complexes embedded in the IMM but also to maintain its crucial barrier function.

### PA POOLS POTENTIALLY USED FOR CL PRODUCTION

Cardiolipin biosynthesis begins with PA, a common substrate in triacylglycerol and glycerolipid metabolism. Reflecting this central role, PA can be made in a multitude of ways. Worth mentioning from the outset is that the source of PA used for CL biosynthesis has yet to be experimentally established; given the apparent redundancy, we anticipate that more than one PA-producing pathway will be at play.

*De novo* biosynthesis of PA is catalyzed by glycerophosphate acyltransferases (GPATs) that are localized to the mitochondrial OMM (GPAT1/2; Yet et al., 1993; Lewin et al., 2004; Wang et al., 2007) or ER (GPAT3/4; Chen et al., 2008; Nagle et al., 2008). GPATs transfer acyl groups from acyl-CoA donors to the *sn*-1 position of glycerol-3-phosphate generating lyso-PA (LPA). Whether and how the mitochondrial and ER GPAT isoforms contribute to mitochondrial phospholipid metabolism is currently unresolved. The LPA that is generated on either the OMM or within the mitochondria-associated membrane (MAM, Vance, 1990; Gaigg et al., 1995) subcompartment of the ER can be acylated by LPA acyltransferases (LPAATs). In mammals, there are four LPAAT isoforms that differ in their tissue distribution and acyl-CoA specificities (some LPAATs can additionally acylate other lyso-lipids; West et al., 1997; Aguado and Campbell, 1998; Lu et al., 2005; Schmidt et al., 2010; Prasad et al., 2011; Eto et al., 2014). In addition to the defined LPAATs, other lyso-phospholipid acyltransferases are able to esterify LPA to some degree (Agarwal, 2012).

Another potential source of PA is mitochondrial phospholipase D (MitoPLD), a divergent member of the PLD superfamily localized to the OMM that can hydrolyze CL to PA *in vitro* (Choi et al., 2006). Similar to yeast, mammals can also produce PA *via* the dihydroxyacetone phosphate pathway in the peroxisome (Hajra and Bishop, 1982). All of the discussed pathways of PA synthesis are located outside of, or on the outside of, mitochondria. However, the recent identification of acylglycerol kinase (AGK) in the IMS suggests that PA may be produced inside the mitochondrion (Hung et al., 2014). AGK, previously termed MuLK for multi-substrate lipid kinase (Waggoner et al., 2004), phosphorylates diacylglycerol (DAG) and monoacylglycerol generating PA and LPA (Waggoner et al., 2004; Bektas et al., 2005). AGK activity is modulated by surface charge (via Mg^2+^) and stimulated, in a dose-dependent manner, by CL (Waggoner et al., 2004). Moreover, overexpression of AGK in the human prostate cancer PC-3 model results in increased mitochondrial PA although CL levels are unchanged (Bektas et al., 2005). Thus, whether PA made by AGK inside of the mitochondrion can and does access the downstream CL biosynthetic machinery is unclear.

### CARDIOLIPIN BIOSYNTHESIS

**FIGURE 1:**
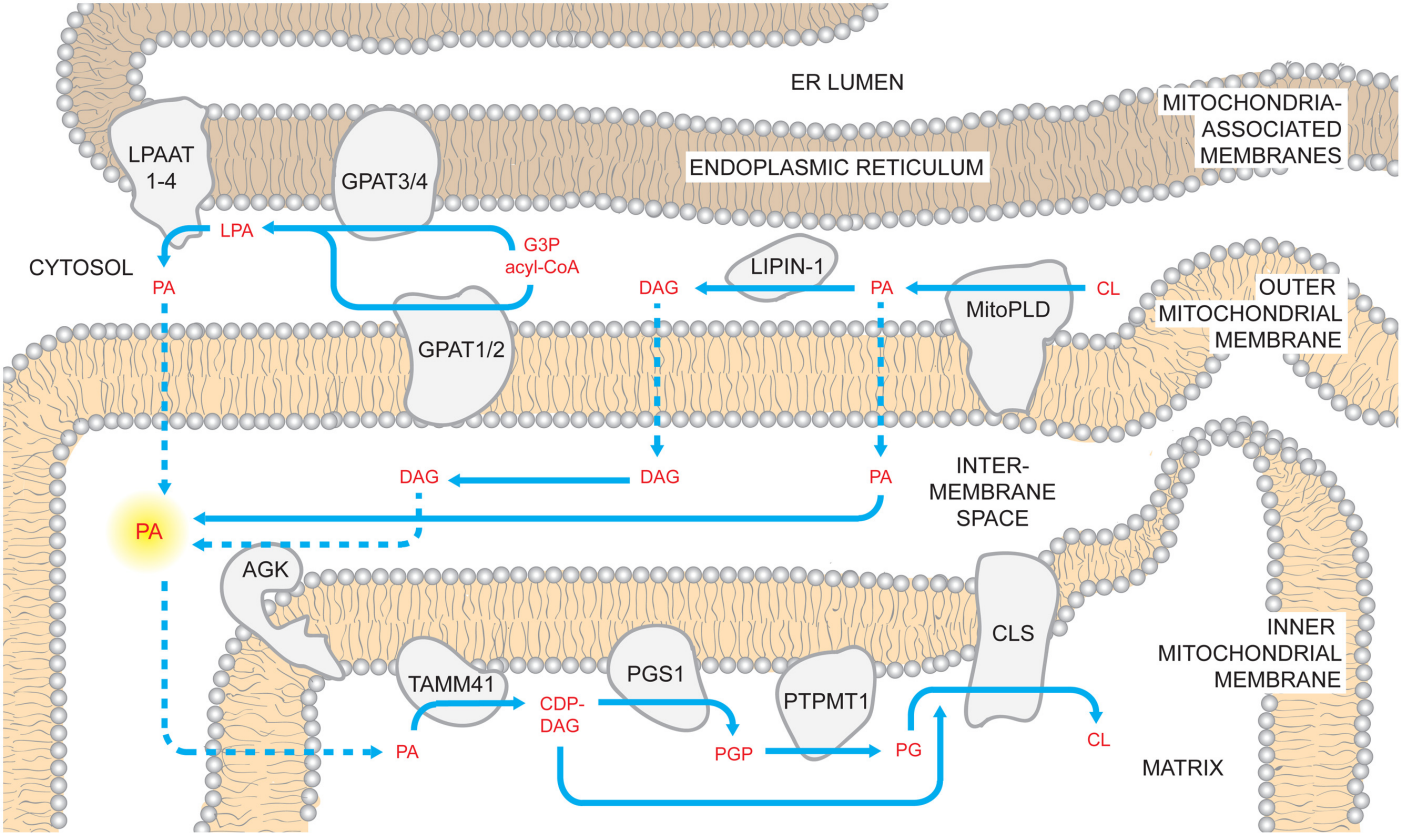
**Mammalian cardiolipin biosynthesis.** CL biosynthesis likely involves PA sourced from multiple pathways. PA can be generated by LPAATs which acylate LPA; LPA can be made using glycerol 3-phosphate (G3P) and acyl-CoAs by mitochondrial and ER GPATs. Additionally, PA can be made on the OMM through the hydrolysis of CL by MitoPLD. In the OMM, PA recruits the phosphatase lipin-1 which dephosphorylates PA into DAG. In turn, DAG may traffic across the OMM and be phosphorylated by AGK forming PA in the context of the IMS-side of the IMM. Regardless of where it is made, PA must reach the matrix side of the IMM to gain access to the core CL biosynthetic machinery. Here, PA is converted to CDP-DAG by TAMM41, thus providing the precursor for the committed step in CL biosynthesis, PGP synthesis by PGS1. PGP is rapidly dephosphorylated by PTPMT1 and the produced PG is condensed with CDP-DAG by CLS generating CL. Dashed arrows describe uncharacterized steps and pathways.

Upon gaining access to the matrix side of the IMM (discussed later), PA is converted to cytidine diphosphate-DAG (CDP-DAG) and pyrophosphate upon reaction with cytidine triphosphate (CTP; **Figure 1**). The reaction is catalyzed by CDP-DAG synthases (CDSs), whose activities have been demonstrated in yeast ER and mitochondria (Kuchler et al., 1986; Shen et al., 1996). It has been recently established that the conserved IMM resident Tam41p is the mitochondrial CDP-DAG synthase whose activity provides CDP-DAG for CL biosynthesis (Tamura et al., 2006, 2013; Kutik et al., 2008). Accordingly, CDS proteins in the ER provide CDP-DAG that is used for phospholipid biosynthesis therein while TAMM41 supplies the CL pathway (Horvath and Daum, 2013).

Also localized on the matrix-facing leaflet of the membrane are downstream enzymes in PG and CL biosynthesis. The committed step of this pathway is catalyzed by phosphatidylglycerol phosphate (PGP) synthase (PGS1), which forms PGP from CDP-DAG and glycerol 3-phosphate (Chang et al., 1998a). After PGP is synthesized, it is rapidly dephosphorylated to PG by PTPMT1 (Xiao et al., 2011; Zhang et al., 2011) of the protein tyrosine phosphatase family that shares no primary sequence similarity to the yeast PGP phosphatase, Gep4p (Osman et al., 2010). Notably, at steady state, PG is present at extremely low levels (1–2%) relative to the other major mitochondrial phospholipids (Daum, 1985), indicating that newly synthesized PG is quickly consumed by downstream pathways. Finally, cardiolipin synthase (CLS), an integral IMM protein with its active site facing the matrix (Schlame and Haldar, 1993), condenses PG with another molecule of CDP-DAG to generate nascent unremodeled CL (Chang et al., 1998b; Chen et al., 2006; Lu et al., 2006).

### CARDIOLIPIN REMODELING

Cardiolipin biosynthetic enzymes exhibit no or only limited acyl chain specificity (Chang et al., 1998b; Chen et al., 2006; Houtkooper et al., 2006). The general lack of acyl chain specificity in CL biosynthesis is significant as it is in direct contrast to the observation that in any given tissue [except the brain (Cheng et al., 2008)], there exists a dominant homogeneous molecular form of CL that is characterized by the incorporation of unsaturated fatty acyl chains (Schlame et al., 1993) and molecular symmetry with respect to the two chiral centers of CL (Schlame et al., 2005). In mammals, CL is predominantly composed of unsaturated 18-carbon linoleic acid (18:2). The enrichment in this particular species is exemplified by human heart where 18:2-linoleic acid constitutes up to 90% of the acyl chains in CL, yielding the abundant and stereotypical tetralinoleoyl-CL species (Schlame and Otten, 1991; Schlame et al., 2005). However, it is notable that the final acyl chain composition of CL in different tissues is not the same; this observation has led to the hypothesis that the final acyl chain composition of CL is tailored to the unique demands of its host tissue (Schlame et al., 2005; Han et al., 2006; Cheng et al., 2008).

**FIGURE 2:**
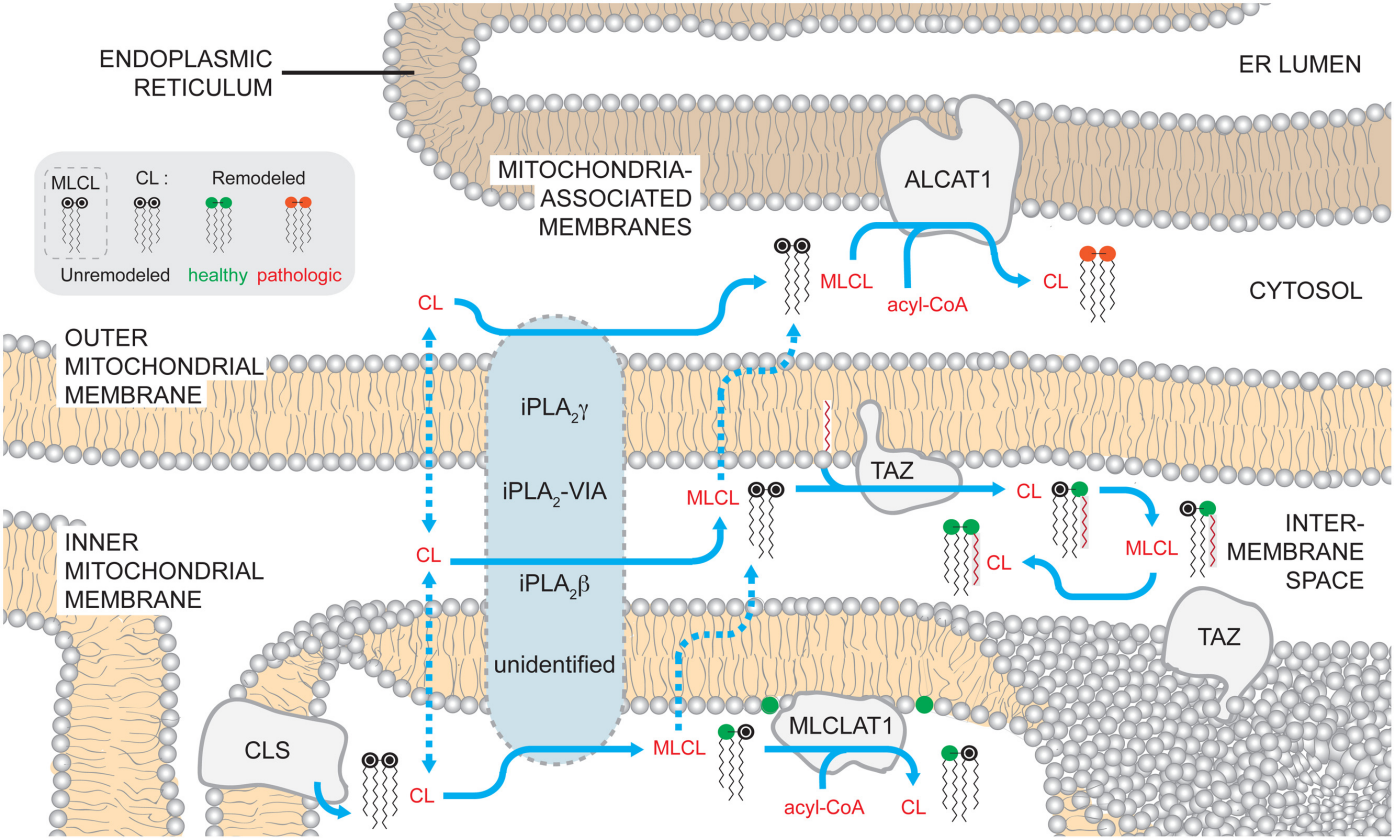
**Mammalian cardiolipin remodeling.** CL that is synthesized by CLS on the matrix-facing leaflet of the IMM can by remodeled by three different pathways. While no single enzyme has been demonstrated to be required to initiate CL remodeling in mammals, several phospholipases of the iPLA_2_ family have been demonstrated to have a role in the process. Additionally, the submitochondrial localization of the phospholipases and the mechanisms by which CL gains access to these enzymes are unknown. After a fatty acyl chain is hydrolyzed from CL, generating MLCL, MLCL can be acylated back to CL by acyltransferases or transacylases. MLCLAT1 on the matrix-leaflet of the IMM, TAZ on the IMS-facing leaflets of the OMM and IMM, and ALCAT1 on the ER MAM all have the capacity to re-acylate MLCL. The acyltransferases, ALCAT1 and MLCLAT1, use acyl-CoAs as an acyl chain donor to acylate MLCL. In contrast, the transacylase TAZ uses acyl chains donated from other phospholipids. The activity of TAZ is required to establish the steady state physiological CL molecular form. In contrast, the CL formed by ALCAT1 is more sensitive to oxidative damage and associated with pathologic states. Dashed arrows describe uncharacterized steps and pathways. In the phospholipid key, unremodeled CL corresponds to the newly synthesized CL that enters the pathway at the point of CLS (black head group). CL can undergo either physiologically relevant CL remodeling (green head group) or pathological remodeling (red head group).

As such, the generation of CL molecular species that accumulate at steady state requires the active remodeling of CL shortly after its *de novo* synthesis (Eichberg, 1974; Schlame and Rustow, 1990). To initiate the remodeling process, a lipase removes an acyl chain from CL generating monolyso-CL (MLCL) that is subsequently re-acylated by one of several enzymes (**Figure 2**). Through a series of such reactions at each position in CL, a tissue-specific homogeneous pool of CL is generated that is characterized by molecular symmetry and a higher degree of acyl chain unsaturation.

#### Phospholipases

In yeast, the recently identified cardiolipin deacylase, Cld1p, initiates CL remodeling and preferentially catalyzes the hydrolysis of palmitic acid (16:0) from newly synthesized CL, forming MLCL (Beranek et al., 2009; Baile et al., 2014b). In the absence of Cld1p, the acyl chain composition of CL shifts to palmitic acid (16:0) residues at the expense of palmitoleic (16:1) and oleic (18:1) acid moieties (Beranek et al., 2009; Baile et al., 2014b). Cld1p associates with the matrix-facing leaflet of the IMM and lacks any membrane spanning segments (Baile et al., 2013), placing the initiation of CL remodeling on the same side of the IMM as its biosynthesis.

Although there are no orthologs of Cld1p in higher eukaryotes, evidence points to Ca^2+^-independent phospholipases A_2_ (iPLA_2_) as being involved in mammalian CL remodeling. The PLA_2_ family of enzymes catalyzes the hydrolysis of membrane glycerophospholipids at the *sn*-2 position, generating free fatty acids and lyso-lipids. Interestingly, all of the PLA_2_ isoforms capable of hydrolyzing CL *in vitro* have different specificities for CL molecular species (Hsu et al., 2013).

iPLA_2_γ is membrane-bound, dually localized to the mitochondrion and peroxisome, and participates in CL metabolism (Mancuso et al., 2000). In the hearts and skeletal muscle of *ipla_*2*_γ^-/-^* mice, CL levels are reduced and the acyl chain pattern is altered (Mancuso et al., 2007; Yoda et al., 2010). However, the absence of iPLA_2_γ in *TAZ* knockdown mice does not prevent the accumulation of MLCL as predicted if iPLA_2_γ functions immediately upstream of TAZ (Kiebish et al., 2013), the major physiological CL remodeling enzyme.

Ablation of another mitochondrially localized iPLA_2_ family member, iPLA_2_-VIA, in *taz*^-/-^ flies, partially restores MLCL:CL ratios, a biochemical hallmark of TAZ dysfunction, and rescues male sterility (Houtkooper et al., 2009a,b; Malhotra et al., 2009a). Still, the CL acyl chain pattern in single *ipla_*2*_-via*^-/-^ flies is not significantly different from wild type (wt) flies, suggesting that like murine iPLA_2_γ, iPLA_2_-VIA activity is not obligately required to initiate the remodeling process. Thus, which phospholipase(s) functions upstream of mammalian TAZ remains an open and important question. Once identified, basic biochemical and cell biological characterization will further establish the topology of CL remodeling. For instance, it is anticipated that the lipase(s) that initiates CL remodeling will reside in the same compartment as the enzyme that subsequently re-acylates MLCL; it is worthwhile to note that although iPLA_2_γ localizes to the mitochondrion, its submitochondrial distribution has not been formally demonstrated. If the lipase and its substrate do not co-localize, then appropriate trafficking steps of the lipid substrate (CL or MLCL) will be inferred. A consideration of the basic cell biology of the lipase(s) required for CL remodeling is particularly relevant given that all three enzymes that can re-acylate MLCL reside in distinct cellular compartments.

#### Trans/acyltransferases

In mammals, there are at least three enzymes that have the capacity to re-acylate MLCL: TAZ, MLCL acyltransferase 1 (MLCLAT1), and acyl-CoA:lysocardiolipin acyltransferase-1 (ALCAT1), of which only TAZ is evolutionarily conserved from yeast to higher eukaryotes (Taylor and Hatch, 2003, 2009; Xu et al., 2006b; Claypool and Koehler, 2012). All evidence to date indicates that the MLCL transacylase, TAZ, is responsible for the vast majority of physiological CL remodeling. TAZ deficiency is biochemically characterized by increased MLCL, the remodeling intermediate, decreased CL, both the substrate and product, and an abnormal acyl chain pattern of the remaining CL (Vaz et al., 2003; Gu et al., 2004; Valianpour et al., 2005; Claypool et al., 2006, 2011; Houtkooper et al., 2009a). However, the mechanism by which TAZ establishes the steady state CL acyl chain composition is unresolved; TAZ catalyzes a reversible transacylation that exhibits no intrinsic acyl chain specificity, acts on acyl chains at both *sn*-1 and *sn*-2 positions, and can use any number of phospholipids and their lyso-derivatives as fatty acyl donors and acceptors, respectively (Xu et al., 2006b; Malhotra et al., 2009b). Notably, heterologous expression of human TAZ in *taz*^-/-^ flies or Δ*taz1* yeast generates CL with acyl chain patterns typical of *Drosophila melanogaster* and yeast, respectively, and not humans (Vaz et al., 2003; Xu et al., 2009). This suggests that the characteristic fatty acid profile of CL may not be conferred by the substrate specificity of TAZ. Interestingly, recombinant fly TAZ can generate the physiologically relevant tetralinoleoyl-CL species from MLCL but only under experimental conditions that promote non-bilayer membranes (Schlame et al., 2012a). This suggests that unique membrane physical states such as regions of high curvature, can confer upon TAZ acyl chain specificity. Other mechanisms that are hypothesized to play a role in the TAZ-based establishment of CL molecular species include the specificity of enzymes immediately upstream of TAZ that initiate the remodeling process and thus dictate which substrates are available to TAZ (Baile et al., 2014b), as well as the dietary intake of fatty acids (Stavrovskaya et al., 2013).

Presently, the submitochondrial localization of endogenous mammalian TAZ has not been documented. Yeast Taz1p associates with the IMS-facing leaflets of the OMM and the IMM as an interfacial protein (Brandner et al., 2005; Claypool et al., 2006; Gebert et al., 2009). This is in sharp contrast to the deacylase, Cld1p that initiates CL remodeling and is localized to the matrix-facing IMM leaflet (Baile et al., 2013). Topologically, this means that MLCL produced on the matrix side of the IMM in yeast has to flip across the membrane and/or be transported to the OMM, to access TAZ for remodeling (Baile et al., 2014a). As TAZ is the only CL remodeling enzyme that is conserved in eukaryotes, it is anticipated that mammalian TAZ will be similarly localized in the organelle. Therefore, determining the submitochondrial localization of the upstream phospholipase in mammals will establish the CL and MLCL trafficking steps needed to access TAZ for remodeling (Baile et al., 2014a).

In mammals, MLCLAT1, which associates with the matrix side of the IMM (Carpenter et al., 1992), does not encounter the same problem of substrate access as TAZ. MLCLAT1 was originally identified in porcine liver mitochondria as a 74-kDa protein (Taylor and Hatch, 2003) that corresponds to the COOH terminus of the human trifunctional protein (TFP) alpha (α) (Taylor and Hatch, 2009). The TFP complex consists of α- and β-subunits that catalyze the last three steps of mitochondrial long-chain fatty acid beta-oxidation thus providing a significant source of cellular energy (Carpenter et al., 1992; Uchida et al., 1992). Pathogenic mutations in either TFP subunit are associated with beta-oxidation disorders, where patients suffer from cardiomyopathy and skeletal myopathy (IJlst et al., 1994; Sims et al., 1995; Ushikubo et al., 1996). Interestingly, recombinant TFPα is soluble when expressed alone, in contrast to its partner TFPβ, suggesting that it may contain a function independent of its association with TFPβ that may be described by MLCLAT1 (Fould et al., 2010). Presently, the gene encoding MLCLAT1 has not been definitively determined. That MLCLAT1 is likely a splice variant of TFPα is suggested by the fact that knockdown using RNAi targeting the NH_2_-terminal portion of TFPα, absent in MLCLAT1, reduces TFPα, but not MLCLAT1 mRNA levels (Taylor et al., 2012). However, whether RNAi targeting a shared region between TFPα and MLCLAT1 can reduce the expression of both genes has not been established.

Both recombinant human TFPα and MLCLAT1 can bind MLCL *in vitro* and are able to incorporate linoleoyl-(18:2), oleoyl- (18:1), and palmitoyl-(16:1) CoA into MLCL (Taylor and Hatch, 2009; Fould et al., 2010; Taylor et al., 2012). When either protein is overexpressed in Barth syndrome (BTHS) lymphoblasts, there is increased incorporation of linoleic acid in CL (Taylor and Hatch, 2009; Taylor et al., 2012). However, TFPα overexpression does not generate a CL profile that reflects its *in vitro* specificity. Thus, while TFPα and/or MLCLAT1 can participate in CL remodeling, especially in the absence of TAZ function when the levels of MLCL are high, the exact contribution of this acyltransferase(s) to physiological CL remodeling is unclear.

Finally, ALCAT1 acylates MLCL and dilyso-CL *in vivo* and can use a number of lyso-phospholipids as acyl acceptors *in vitro* (Cao et al., 2004, 2009; Zhao et al., 2009). Depending on the acyl acceptor, tissue type, and species, ALCAT1 has been described to preferentially incorporate long-chain unsaturated fatty acyl chains or promiscuously accept all acyl-CoA derivatives (Cao et al., 2004; Zhao et al., 2009). As such, ALCAT1 lacks the specificity expected of an enzyme with a critical role in physiological CL remodeling. Notably, ALCAT1 resides in the ER MAMs (Cao et al., 2004; Zhao et al., 2009). Therefore, for ALCAT1 to participate in CL metabolism, CL and/or MLCL must travel from the IMM to at least the external leaflet of the OMM, if the active site of ALCAT1, which has not been determined, faces the cytosol.

The absence of TAZ causes alterations in CL molecular species in every model tested to date (Vreken et al., 2000; Bissler et al., 2002; Valianpour et al., 2002, 2005; Schlame et al., 2003, 2005; Vaz et al., 2003; Gu et al., 2004; Xu et al., 2005, 2006a,b; van Werkhoven et al., 2006; Acehan et al., 2009, 2011b; Houtkooper et al., 2009a,b; Dudek et al., 2013; Gonzalvez et al., 2013; Baile et al., 2014b; Wang et al., 2014a). In contrast, the loss of MLCLAT1/TFPα or ALCAT1 does not consistently result in changes in the steady state acyl chain composition of CL (Li et al., 2010; Schlame et al., 2012b; Richter-Dennerlein et al., 2014). Thus, it is unlikely that either MLCLAT1/TFPα or ALCAT1 significantly contributes to the steady state CL acyl chain profile under normal conditions. These results further underscore the predominant role of TAZ in physiological CL remodeling.

### PHOSPHOLIPID TRAFFICKING STEPS REQUIRED FOR CL METABOLISM

**FIGURE 3:**
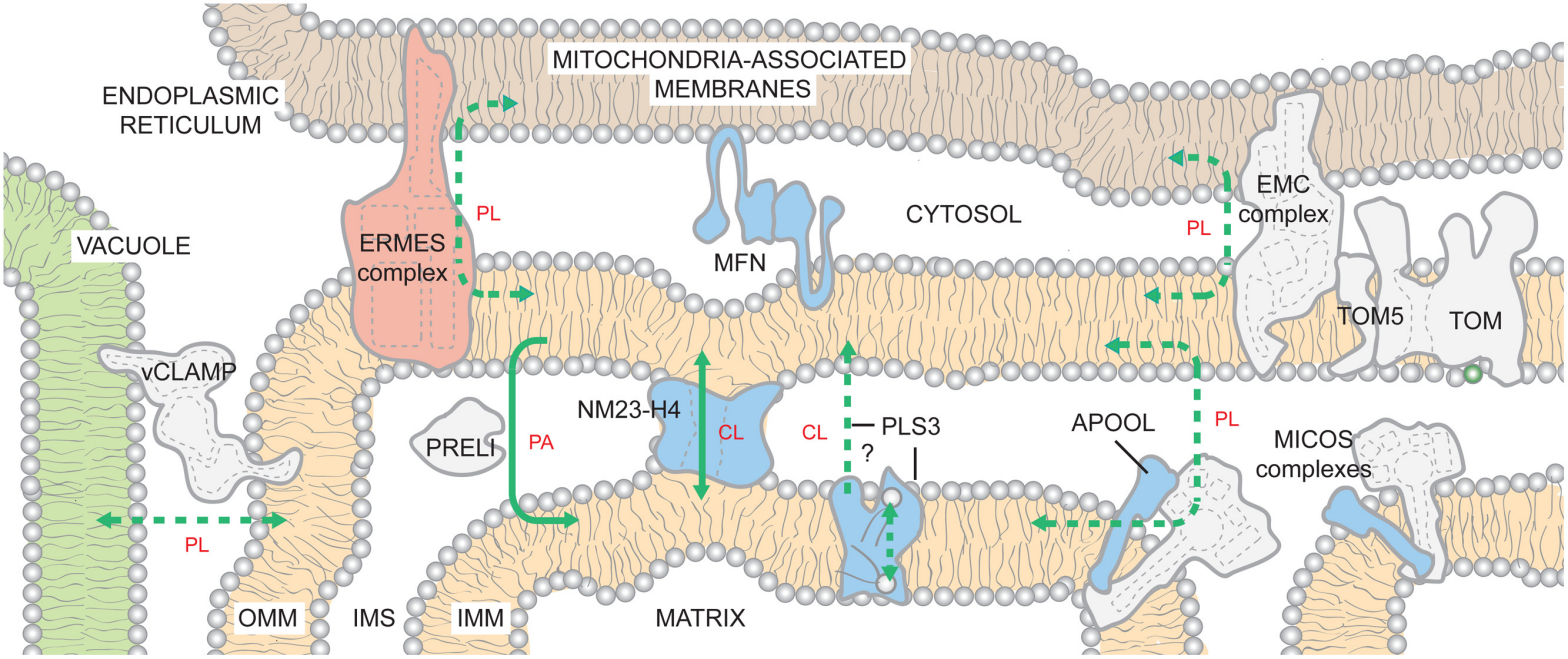
**Inter-organelle and intra-organelle phospholipid trafficking.** The existence of ER- and vacuole-mitochondria contacts is highly conserved from yeast to humans. By generating closely appositioned membranes, the inter-organelle and intra-organelle tethers are hypothesized to promote movement of lipids across the aqueous cytosol and IMS, respectively. Within the mitochondrion, phospholipid trafficking may involve contacts between the OMM and the IMM mediated by MICOS complexes or NM23-H4. In addition, PRELI transports PA from the OMM to the IMM. PLS3 activity stimulates CL externalization on OMM. It may directly transport CL from the IMM to the OMM or instead function as a scramblase that redistributes CL between both leaflets of the IMM. CL now exposed to IMS-side of IMM would then be transported to the OMM by other mechanisms. With the possible exception of EMC, it is presently unclear if any of the known tethers has specificity for a defined phospholipid(s); as such, they are shown to promote the flux of phospholipids (PLs) in bulk. If a specific phospholipid is impacted by mutations in a complex/protein (levels and/or composition), the lipid is indicated. Solid lines indicate known transport mechanisms. Dashed lines describe possible trafficking routes and/or highlight transport events whose mechanisms have not been resolved. The ERMES complex is found only in yeast and color-coded pink. For the remaining proteins/complexes, those found only in mammals are in blue and those that are likely to be conserved across species are colored gray. See text for additional details.

Once made, PA must travel to the matrix side of the IMM to gain access to the CL biosynthetic machinery. If made in the ER, this requires movement of PA from ER to OMM, flipping between OMM leaflets, movement from the OMM to the IMM, and finally flipping to the matrix-facing IMM leaflet. Inter-organelle contacts have recently emerged as being critically important for mitochondrial phospholipid metabolism. In yeast, there are at least two distinct structures that contribute to the physical association of ER and mitochondria (**Figure 3**). The first described complex is the ER-mitochondria encounter structure (ERMES; Kornmann et al., 2009). A second ER-mitochondria tether, the ER-membrane protein complex (EMC), that is distinct from ERMES, was recently identified in yeast (Lahiri et al., 2014). When either EMC or ERMES subunits are missing, the number and length of ER-mitochondria contacts are reduced and mitochondria are unable to support growth on respiratory media (Kornmann et al., 2009, 2011; Tamura et al., 2012; Lahiri et al., 2014). Also, a synthetic mitochondria-ER tether similarly rescues the defects caused by the absence of EMC or ERMES. While EMC may play a more direct role in phospholipid trafficking (Lahiri et al., 2014), EMC and ERMES complexes likely have both overlapping and distinct functions in phospholipid transfer between the ER and mitochondria (Nguyen et al., 2012; Lahiri et al., 2014).

The ERMES subunits are conserved in fungi but not in metazoans. In contrast, the close apposition of ER and mitochondria is conserved (Csordas et al., 2006). In mammals, IP_3_ receptor/GRP75/VDAC1-containing complexes (Matsuzaki et al., 2013), mitofusin-2 (de Brito and Scorrano, 2008), and several other proteins have roles in ER-mitochondria tethering (Merkwirth and Langer, 2008; Csordas et al., 2010). Moreover, the newly described EMC is conserved but its role in ER-mitochondrion tethering has only been tested in yeast. The diversity of players implicated in this inter-organelle association provides strong evidence of the physiological importance of such contacts.

The existence of vacuolar and mitochondrial contacts, termed vCLAMPs (vacuole and mitochondrial patch), recently identified in yeast, further highlights the importance of inter-organelle associations for mitochondrial phospholipid metabolism (Elbaz-Alon et al., 2014; Honscher et al., 2014). Simultaneous deletion of ERMES and vCLAMP components is synthetically lethal. Deletion of vCLAMP and repression of ERMES impairs PA trafficking into mitochondria and results in a 40% reduction in CL levels (Elbaz-Alon et al., 2014). A recent report describing mitofusin-2-mediated contacts between mouse melanosomes (a lysosomal-like compartment in pigment cells) and mitochondria (Daniele et al., 2014), suggests that there may be similar vacuolar-tethering mechanisms in mammals.

Analogous to inter-organelle tethers, regions of close physical apposition between the IMM and OMM are important for lipid trafficking across the IMS (Ardail et al., 1990; Schlattner et al., 2014), although the underlying mechanism(s) is unknown. In addition, IMM-OMM contact sites have roles in energy transduction from the mitochondrial matrix to the cytosol (Nicolay et al., 1990) and precursor protein import (Vogel et al., 2006). MICOS (mitochondrial contact site and cristae organizing system) is a hetero-oligomeric protein complex that is embedded in the IMM but interacts with several distinct OMM proteins (Harner et al., 2011; Hoppins et al., 2011; von der Malsburg et al., 2011; van der Laan et al., 2012; Pfanner et al., 2014). Depletion of the conserved mitofilin/Fcj1p (yeast formation of cristae junction 1) subunit, which constitutes the MICOS core, results in an expanded IMM surface, dramatic loss of cristae junctions, and the remaining cristae are stacked, lamellar, and aberrantly disconnected from the OMM (John et al., 2005; Mun et al., 2010; Harner et al., 2011; von der Malsburg et al., 2011). Recently, apolipoprotein O (APOO) and APOO-like protein (APOOL) were identified as potential subunits of the bovine MICOS complex (Weber et al., 2013). The IMS-facing APOOL specifically binds CL *in vitro* and its knockdown results in morphological phenotypes similar to yeast MICOS mutants (Weber et al., 2013). Thus, MICOS complexes are key determinants of cristae morphology that contain subunits capable of binding CL (Harner et al., 2011; Hoppins et al., 2011; von der Malsburg et al., 2011). Whether these two properties are leveraged in the context of phospholipid trafficking between the IMM and OMM is presently unclear although it is interesting to note that biochemically isolated IMM-OMM contact sites are enriched in CL (Ardail et al., 1990; Simbeni et al., 1991).

The movement of PA from the OMM to the IMM is mediated by the IMS-resident Ups1p/PRELI-like proteins (Connerth et al., 2012; Potting et al., 2013). *In vitro*, the yeast Ups1p/Mdm35p dimer (Potting et al., 2010; Tamura et al., 2010) binds numerous anionic phospholipids but only transports PA (Connerth et al., 2012). The directionality of PA transport is likely conferred by the fact that Ups1p remains tightly bound to membranes containing physiological amounts of CL, leading to Ups1p’s subsequent degradation. These observations suggest a mechanism for regulating CL biosynthesis by limiting precursor trafficking between the two membranes when CL levels are bountiful (Connerth et al., 2012). Whether transport of PA by the Ups1p/PRELI-like proteins utilizes in some manner MICOS or is instead mechanistically distinct is at present unknown.

Once PA is transported to the IMM, or if synthesized in this compartment by AGK, the transbilayer movement of PA to the matrix-leaflet is required for PA to gain access to the CL biosynthetic enzymes. How this is achieved is presently not known but could involve a specific protein or protein complex. However, the requirement for a specific PA transporter does not seem obligate as a transmembrane pH gradient is sufficient to disseminate PA across both IMM leaflets (Hope et al., 1989; Gallet et al., 1999).

Since CL is made in the context of the matrix-leaflet and can eventually be exposed on the OMM, mechanisms must be present to facilitate the movement of CL intra-mitrochondrially. Scramblases are Ca^2+^-dependent, ATP-independent bidirectional transporters that equilibrate lipids unevenly distributed across a bilayer (Contreras et al., 2010). As such, a scramblase could serve to redistribute CL made on the matrix side, between IMM leaflets. Alternatively, phospholipid translocation between membrane leaflets may not be mediated by specific proteins, but instead facilitated by the presence of numerous transmembrane proteins (especially in the context of the IMM) in a non-specific manner, as suggested for bacterial and ER membranes (Kol et al., 2001, 2004).

Albeit minor, CL is a normal constituent of the OMM and can traffic to the OMM following certain stimuli (Gonzalvez et al., 2008; Chu et al., 2013). Phospholipid scramblase 3 (PLS3) is the only known mitochondrial scramblase (Zhou et al., 1998; Liu et al., 2003a; Van et al., 2007) and *in vitro*, murine and human PLS3 catalyze the Ca^2+^-dependent flip-flop of CL in proteoliposomes (Zhou et al., 1998). However, it is unclear whether PLS3 functions *in vivo* as a CL scramblase or instead mediates the movement of CL from the IMM to the OMM. PLS3 overexpression increases mitochondrial mass, *CLS* transcription, CL synthesis, and CL externalization to the OMM (Liu et al., 2003b; Van et al., 2007). Conversely, overexpression of a catalytically dead *pls3* allele or *PLS3* knockdown reduces CL externalization following UV irradiation or rotenone poisoning, respectively (Liu et al., 2003b; Chu et al., 2013). How does PLS3 activity contribute to movement of CL to the OMM? If PLS3 is a true scramblase, then the equilibration of CL between the leaflets of the IMM may be required for the subsequent ability of CL to traffic to the OMM. Alternatively, PLS3 may instead directly participate in the movement of CL between mitochondrial membranes. Future studies are needed to clarify the role of PLS3 in this process.

Another potential mechanism by which lipids can be transferred from the IMM to the OMM involves the NM23-H4/NDPK-D (nucleotide diphosphate kinase isoform D; Milon et al., 2000). NM23-H4 is the only mitochondrially targeted member of a family of NDPKs whose role in phosphotransfer is well-established. NM23-H4 has been additionally implicated in the trafficking of anionic phospholipids (in particular CL) between the IMM and the OMM (Tokarska-Schlattner et al., 2008). Interestingly, the levels of CL can functionally switch NM23-H4 between phosphotransfer and lipid transfer modes (Schlattner et al., 2013). Normally, the lipid transfer mode is inhibited by anionic lipids, including CL, and the protein operates as a nucleotide kinase. However, when CL levels are low (due to mitochondrial dysfunction), NM23-H4’s lipid transfer function is de-repressed and the protein cross-links the IMS-facing leaflets of the IMM and OMM. Subsequently, NM23-H4 facilitates the thermodynamically unfavorable movement of negatively charged lipids across the aqueous IMS.

While a role for both NM23-H4 and PLS3 in the stimulated externalization of CL on the OMM is clearly emerging, whether either or both enzymes participate in the routine processes of CL biosynthesis and remodeling is not known. Given its MAM-residence, it is tempting to speculate that NM23-H4 and/or PLS3 may be involved in ALCAT1-based CL remodeling.

## PHYSIOLOGICAL FUNCTIONS

Phospholipids play a myriad of roles in cellular and mitochondrial physiology that are beyond the scope of any single review. The following section is focused on recently discovered roles and guided by those physiological functions of mitochondrial lipids, that when disturbed, may contribute to human disease. Of note, the diversity of functions attributed to the discussed mitochondrial phospholipids is reflected by the vast array of pathogenic mechanisms that underlie this cohort of mitochondrial diseases.

### PHOSPHATIDIC ACID

The dynamic appearance and disappearance of PA on the OMM is a recently established determinant of mitochondrial fusion and fission. Overexpressed MitoPLD generates PA on the OMM, promoting mitochondrial fusion and subsequent aggregation (Choi et al., 2006), while loss of MitoPLD leads to fragmented mitochondria (Huang et al., 2011). PA generated by MitoPLD recruits the PA phosphatase, lipin-1. Lipin-1 dephosphorylates PA to DAG which stimulates mitochondrial fission while simultaneously removing the pro-fusogenic accumulation of PA on the OMM (Reue and Dwyer, 2009; Huang et al., 2011). Interestingly, *mitopld^-/-^* flies (Pane et al., 2007) and mice (Huang et al., 2011; Watanabe et al., 2011) have defects in the biogenesis of Piwi-interacting (pi)RNAs that have a role in providing a germline-specific defense against retrotransposon activity (Gunawardane et al., 2007). Male *mitopld^-/-^* flies are sterile, typical of flies lacking piRNAs (He et al., 2009), lose nuages (Russell and Frank, 1978), sites where piRNA production and processing is thought to occur, and have de-repressed retrotransposons in their testes (Pane et al., 2007; Watanabe et al., 2011). Moreover, *lipin-1^-/-^* mice have elevated PA on the mitochondrial surface and significantly increased nuage formation (Huang et al., 2011). Genetic evidence therefore strongly supports a role for PA and/or DAG at the OMM in piRNA production (Huang et al., 2011). However, the exact role of MitoPLD with respect to the mitochondrial phospholipid pool is unclear.

The importance of the dynamic regulation of PA on the OMM is further substantiated by the recent characterization of PA-PLA_1_ (Baba et al., 2014). *In vitro*, PA-PLA_1_ preferentially deacylates PA to LPA (Higgs and Glomset, 1994). PA-PLA_1_ overexpression or depletion in HeLa cells causes mitochondrial fragmentation and elongation, respectively (Baba et al., 2014). Interestingly, co-expression of PA-PLA_1_ and MitoPLD prevents the accumulation of PA on the OMM surface and the morphological defects associated with MitoPLD overexpression alone (Baba et al., 2014). Similar to *mitopld^-/-^*and *lipin-1*^-/-^ mice, *pa-pla_1_^-/-^* mice have a defect in spermatogenesis that correlates with mitochondrial disorganization (Baba et al., 2014). Finally, diminution of *ddhd2*, a related iPLA_1_ family member with a similar specificity for PA as PA-PLA_1_, causes mitochondrial elongation in mouse embryonic fibroblasts (Baba et al., 2014).

### PHOSPHATIDYLGLYCEROL

Besides being a required intermediate in CL biosynthesis, PG is also a precursor for bis(monoacylglycerol)phosphate (BMP; Hullin-Matsuda et al., 2007), a class of phospholipid that is highly enriched in late endosomes and lysosomes (Wherrett and Huterer, 1972; Poorthuis and Hostetler, 1976; Mobius et al., 2003). While BMP is found in many tissues and cells, it is usually present at less than 1% of the total phospholipid mass (Simon and Rouser, 1969; Mason et al., 1972). BMP is a structural isomer of PG and is thought to function in the maintenance and regulation of endosomal/lysosomal membrane dynamics and cholesterol trafficking (Kobayashi et al., 1998, 1999; Hullin-Matsuda et al., 2009; Gallala and Sandhoff, 2011). However, its exact biological role(s) is unresolved.

### CARDIOLIPIN

**FIGURE 4:**
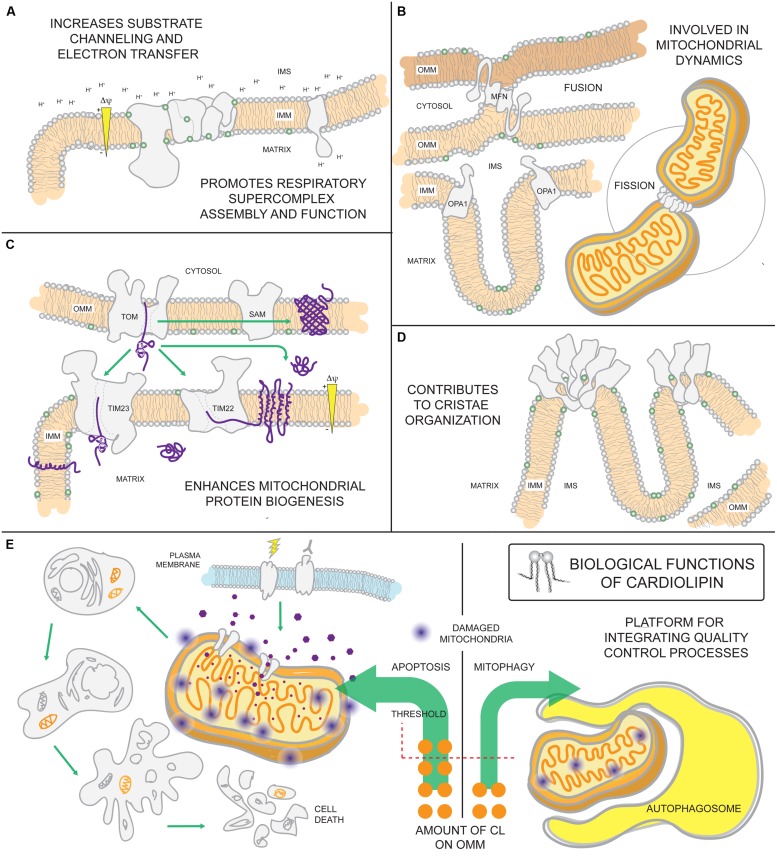
**Biological functions of cardiolipin.** As the signature phospholipid of the mitochondrion, CL is intimately involved in a number of mitochondrial processes. **(A)** Anionic CL on the IMM can function as a proton trap by attracting (and providing) a local pool of protons that can be funneled towards the ATP synthase. Moreover, CL is associated with every OXPHOS component and can promote their assembly into respiratory supercomplexes. Such supramolecular assemblies are thought to enhance electron transfer and reduce ROS leakage from the electron transport chain. **(B)** CL associates with dynamin-related GTPases that are intimately involved in fusion and fission and **(C)** contributes to the assembly and function of IMM and OMM translocases vital for mitochondrial biogenesis. **(D)** Besides enhancing OXPHOS by stabilizing SCs, CL also promotes the assembly of ATP synthase oligomers that provide a structural scaffold required for establishing the characteristic shape of mitochondrial cristae. **(E)** Externalization of CL on the surface of the mitochondrion is involved in signaling the execution of either mitophagy or apoptotic cell death.

The absolute requirement of PG and/or CL for life is underscored by the observation that *ptpmt1*^-/-^ mice die *in utero* before embryonic day 8.5 (Zhang et al., 2011). Reflecting this importance, CL has a multitude of functional roles in mitochondria (**Figure 4**). CL is highly enriched in cardiac tissues making up 15–20% of the total phospholipid phosphorus mass of the heart (Pangborn, 1942; Hostetler et al., 1975). Its relative abundance in cells and tissues with high energetic demands point to CL as being intimately involved in maintaining mitochondrial structure and function. Indeed, it interacts with numerous mitochondrial proteins, including all OXPHOS complexes and most mitochondrial solute carriers, and is often required for their functional reconstitution in liposomes (Cheneval et al., 1983; Beyer and Klingenberg, 1985; Eble et al., 1990; Shinzawa-Itoh et al., 2007; Claypool et al., 2008; Claypool, 2009; Schwall et al., 2012). In addition, CL is proposed to function as a proton trap that helps funnel pumped protons toward the ATP synthase to generate ATP (Haines and Dencher, 2002). An association with CL promotes the assembly of membrane proteins into oligomeric complexes (Zhang et al., 2002; Pfeiffer et al., 2003; Claypool et al., 2008; Strauss et al., 2008; Acehan et al., 2011a; Althoff et al., 2011). Indeed, CL is important for the assembly and function of IMM and OMM translocases and thus, mitochondrial biogenesis (Jiang et al., 2000; van der Laan et al., 2007; Gebert et al., 2009). CL is also critically important for stabilizing respiratory supercomplexes (SCs), supramolecular assemblies built from respiratory complexes I, III, and IV (Schagger and Pfeiffer, 2000). These SCs are thought to increase the efficiency of electron transfer between the respiratory chain components by substrate channeling mechanisms (Acin-Perez et al., 2008; Lapuente-Brun et al., 2013), thereby maximizing OXPHOS. Further, SC assembly shortens the distance traveled by mobile electron carriers, minimizing ROS leakage and reducing oxidative damage. Finally, CL-binding stimulates the activity of dynamin-related GTPases with pivotal roles in IMM fusion and mitochondrial fission (Ban et al., 2010; Montessuit et al., 2010; Bustillo-Zabalbeitia et al., 2014). All of these CL-supported functions have been discussed extensively in several excellent reviews (Chicco and Sparagna, 2007; Houtkooper and Vaz, 2008; Joshi et al., 2009; Lewis and McElhaney, 2009; Sparagna and Lesnefsky, 2009; Osman et al., 2011; Claypool and Koehler, 2012).

Both apoptosis and mitophagy, a macro-autophagic process that is pre-emptive to cell death *via* apoptosis, are signaled in part by the externalization of CL to the OMM. The movement of CL to the OMM is thought to involve the scramblase PLS3 and/or NM23-H4 and may occur preferentially at IMM-OMM contact sites thought to be enriched in CL (Ardail et al., 1990; Simbeni et al., 1991). Overexpression of NM23-H4, but not a CL-binding mutant, increases apoptotic markers that may be ascribed to increased CL externalization on the OMM (Schlattner et al., 2013). CL on the OMM attracts and activates caspase-8 (Gonzalvez et al., 2008) and aids in pro-apoptotic Bax insertion into and permeabilization of the OMM (Kuwana et al., 2002; Lucken-Ardjomande et al., 2008). In preparations of giant unilamellar vesicles lacking CL, caspase-8 is unable to interact with vesicle membranes, a step necessary for caspase-8 activation and subsequent recruitment of tBid (Jalmar et al., 2010, 2013). Yeast mitochondria lacking CL are protected from the bioenergetic perturbations normally induced upon incubation with tBid (Gonzalvez et al., 2005) underscoring the importance of CL for tBid function. Furthermore, CL peroxidation by cytochrome c promotes the release of a number of pro-apoptotic factors, including cytochrome c, following OMM permeabilization (Kagan et al., 2005). Under conditions of mild mitochondrial dysfunction, CL is externalized on the OMM where it promotes the specific destruction of the mitochondrion by mitophagy (Chu et al., 2013). siRNA knockdown of the scramblase PLS3 or CL synthase CLS in neuronal cells, diminishes CL externalization and the number of mitochondrially-associated autophagic markers, and attenuates chemically-induced mitophagy (Chu et al., 2013). As CL externalization is important for both apoptosis and mitophagy, there is likely to be a threshold level of mitochondrial damage (severity of insult and percentage of mitochondrial pool impacted) above which apoptosis is executed and below which the affected mitochondria are selectively removed. In addition, qualitative and/or quantitative differences in the CL exposed on the OMM may influence how this lipid signal is interpreted by the cell.

### REMODELED VERSUS UNREMODELED CL

While the role and functional consequence of MLCAT1-based remodeling is presently unresolved, TAZ- and ALCAT1-mediated CL remodeling are associated with very different physiologic outcomes. In the absence of TAZ, the acyl chain composition of CL is significantly diversified and molecular symmetry is lost (Schlame et al., 2005). Thus, TAZ has a preeminent role in dictating the final collection of acyl chains attached to CL under physiological conditions. The final acyl chain pattern of CL, which is tissue-specific, is thought to be critical for normal mitochondrial physiology by supporting some combination of the functions attributed to CL. However, Δ*cld1* yeast, which fail to initiate CL remodeling and accumulate normal amounts of unremodeled CL, have wt OXPHOS activity and normal mitochondrial morphology (Baile et al., 2014b; Ye et al., 2014a). These results question the idea that TAZ-based CL remodeling produces “optimized” CL species that promote mitochondrial fitness, and instead suggests that CL remodeling may actually accomplish other physiologically important functions that either were not tested (Baile et al., 2014b; Ye et al., 2014a) or have not yet been discovered.

In contrast to TAZ-based CL remodeling, CL remodeled by ALCAT1 predisposes mitochondria to damage. In mouse myoblasts, ALCAT1 overexpression increases the amount of CL enriched with docosahexaenoic acid (22:6) at the expense of linoleic acid (18:2; Li et al., 2010), consistent with alterations in CL species that are observed in aging rat hearts [the latter additionally contain 20:4 arachidonic acid (Paradies et al., 2009)]. The increased acyl chain unsaturation in these CL forms makes them peroxidation-prone and increases the susceptibility of mitochondria to undergo apoptosis (Watkins et al., 1998; Paradies et al., 2009; Li et al., 2010). As such, ALCAT1 seems to perform “pathogenic” remodeling of CL. Consistent with this, ALCAT1 overexpression, also noted in mouse models of metabolic disease, increases the rate of ATP, and consequently, ROS production during oxidative stress (Li et al., 2010). Conversely, ablation of ALCAT1 elevates tetralinoleoyl-CL content in the heart (Li et al., 2010), prevents the onset of disease, increases insulin resistance, mitigates OXPHOS dysfunction by increasing complex I activity, restores mtDNA fidelity, alleviates fusion defects and associated mitochondrial fragmentation, and re-establishes mitochondrial quality control (Li et al., 2010, 2012; Liu et al., 2012; Wang et al., 2014b). Combined, this suggests that CL remodeled by ALCAT1 may exacerbate and/or signal mitochondrial dysfunction in disease pathogenesis (Wang et al., 2014b).

## EMERGING DISEASES OF MITOCHONDRIAL PHOSPHOLIPID METABOLISM

With the recent application of next generation sequencing methodologies, new disease-causing genes are being implicated in mitochondrial disorders each year. For the remainder of this review, we describe a new category of mitochondrial disorder that is caused by nuclear defects that specifically alter mitochondrial phospholipid metabolism. We anticipate that the diseases discussed below represent the tip of the iceberg and that more disorders that impinge on mitochondrial phospholipid metabolism will be identified in the near future.

### TAZ MUTATIONS LEADING TO BARTH SYNDROME

Mutations in the gene that encodes the MLCL transacylase, TAZ, lead to BTHS (Barth et al., 1983, 2004; Bione et al., 1996). BTHS is the founding member of this new class of mitochondrial disease and thus not surprisingly, is the best characterized. This X-linked multisystem disorder presents with cardiomyopathy, skeletal muscle weakness, neutropenia, growth retardation, and 3-methylglutaconic aciduria (3-MGA), and can be fatal if not diagnosed early [Barth et al., 1983; Kelley et al., 1991; Johnston et al., 1997; one isolated case of BTHS in a female patient has been reported (Cosson et al., 2012)]. 3-MGA is a heterogeneous group of syndromes characterized by an increased excretion of 3-methylglutaconic and 3-methylglutaric acids, breakdown products of leucine catabolism (Wysocki et al., 1976). Additional features such as isolated left ventricular non-compaction, ventricular arrhythmia, motor delay, exercise intolerance, poor appetite, fatigue, hypoglycemia, lactic acidosis, and hyperammonemia have also been described in BTHS patients (Ichida et al., 2001; Steward et al., 2010). Patient heart, liver, and skeletal muscle biopsies contain malformed mitochondria with tightly stacked or circular bundles of cristae (Barth et al., 1983; Hodgson et al., 1987; Orstavik et al., 1998; Bissler et al., 2002). In patient-derived lymphoblasts, mitochondria have dramatically reduced inner membranes, collapsed cristae, and are often fragmented (Xu et al., 2005; Acehan et al., 2007). *TAZ* (G4.5) contains 11 exons, is localized on a gene-rich region on Xq28, and is highly expressed in cardiac and skeletal tissues (Bione et al., 1996). Pathogenic *taz* variants identified to date encompass splice site mutations, insertions, deletions, as well as missense and nonsense mutations (Johnston et al., 1997; a current list of known genetic variants is maintained by the BTHS foundation; ).

Efforts to understand BTHS pathogenesis are complicated by the complete lack of genotype–phenotype correlations. Patients with the same mutation, or even siblings sharing the same mutation, can manifest with extremely disparate symptoms. A case in point is that of a 51 years old proband, the oldest BTHS patient reported, and his 3 years old grandnephew (Ronvelia et al., 2012). Although they harbor the same mutation, the boy presented with cardinal manifestations of BTHS (including congestive heart failure that required a heart transplant at 11 months of age) while the great uncle was 43 years old when he was diagnosed with myopathy. These observations highlight the importance of modifying factors as key determinants in BTHS disease progression.

Furthermore, links between different mutations and severity of disease have not been established for BTHS. Model organisms can help bridge this gap. Using a yeast BTHS model, 21 distinct pathogenic missense mutations have been modeled in yeast Taz1p and their loss-of-function mechanism defined. This effort has identified seven classes of BTHS mutants defined by their loss-of-function mechanisms (Claypool et al., 2006, 2011; Whited et al., 2013). Briefly, the seven classes comprise of variants that are (1) non-functional truncated products resulting from frameshifts or aberrant splicing, (2) mislocalized within mitochondria and aggregation-prone, (3) aberrantly assembled, (4) catalytically dead, (5) hypomorphic alleles with residual transacylase activity, (6) unable to engage in stable productive assemblies, or (7) temperature-sensitive. Systematic analyses of pathogenic variants in this manner can provide important mechanistic insight into the clinical heterogeneity of BTHS. The next step in attaining this goal is to verify that the defined loss-of-function mechanisms are conserved in an appropriate mammalian model. Such a model is additionally needed to characterize pathogenic alleles that cannot be modeled in yeast due to a lack of conservation.

There are a number of BTHS models presently available. Importantly, every BTHS model has the characteristic biochemical defects that underlie BTHS – increased MLCL, decreased CL, and an abnormal acyl chain composition of the remaining CL. In addition to yeast, other cellular BTHS models include patient-derived fibroblasts (Barth et al., 1996), lymphocytes (Schlame et al., 2005; Xu et al., 2005), and iPSCs (Dudek et al., 2013; Wang et al., 2014a), and *TAZ*-depleted rodent (Acehan et al., 2009; He, 2010; He et al., 2014) and human cell lines (Gonzalvez et al., 2008). Animal models of BTHS include *TAZ*-depleted zebrafish (Khuchua et al., 2006) and mice (Acehan et al., 2011b; Soustek et al., 2011; Phoon et al., 2012), and *taz*^-/-^ flies (Xu et al., 2006a).

Cellular models of BTHS show dysmorphic changes in mitochondrial morphology and energetic defects (Acehan et al., 2007, 2009; Gonzalvez et al., 2013). In patient-derived lymphoblasts (Xu et al., 2005; Gonzalvez et al., 2013), iPSCs (Dudek et al., 2013), and fibroblasts (Barth et al., 1996), there is low basal respiration, reduced membrane potential, and compromised coupling of OXPHOS. Respiratory SCs are decreased and there is a shift in SC assembly from large “respirasomes” to smaller, and presumably, less efficient SCs (McKenzie et al., 2006; Dudek et al., 2013; Gonzalvez et al., 2013). Therefore, it is postulated that these alterations in respiratory chain assembly diminish respiratory efficiency and consequently, augment ROS production. While basal respiration is unaffected in the shRNA-inducible *TAZ* knockdown mouse, maximal uncoupled respiration is reduced; whether this reflects changes in respiratory SC stability has not been demonstrated (Powers et al., 2013). Enzymatic analyses of respiratory chain complexes using cardiomyocytes derived from the *TAZ* knockdown mouse indicate that complex III is impaired (Powers et al., 2013), suggestive of a similar bioenergetic dysfunction as noted in the cellular models. In *TAZ*-depleted mice (Acehan et al., 2011b; Soustek et al., 2011; Phoon et al., 2012) and zebrafish (Khuchua et al., 2006), cardiac defects are observed that recapitulate many of the relevant cardiac parameters noted in BTHS patients. With respect to zebrafish, *TAZ* knockdown severely impairs zebrafish development and the degree of cardiac dysmorphology is proportional to the morpholino dose. In addition to impaired cardiac function, *TAZ* knockdown mice have abnormal skeletal muscle ultrastructure (Xu et al., 2006a; Acehan et al., 2011b) consistent with *taz^-/-^* flies that also have impaired muscle functions (Xu et al., 2006a; Acehan et al., 2009). Recently, cardiomyocytes differentiated from BTHS patient iPSCs have been generated (Dudek et al., 2013; Wang et al., 2014a). In these cells, there is structural destabilization of respiratory SCs that correlates with reduced respiratory complex activities (Dudek et al., 2013). When wt cells are seeded onto engineered chips, they form sarcomeres and contract; in contrast, the ability of BTHS-derived cardiomyocytes to form organized sarcomeric arrays is severely impacted as is their contractility (Wang et al., 2014a). Therefore, results from the myriad of BTHS models indicate that the lipid abnormalities that occur in the absence of TAZ result in OXPHOS dysfunction associated with SC destabilization. OXPHOS dysfunction increases the production of ROS and in sum, these impairments compromise heart development and function.

As already discussed, the stimulated externalization of CL onto the OMM is an important event that can alternatively trigger apoptosis or mitophagy. It is thus notable that BTHS-derived lymphocytes are resistant to mitochondrial-dependent apoptosis due to an impaired ability to recruit and activate caspase-8 (Gonzalvez et al., 2008, 2013). Interestingly, *caspase-8*^-/-^ mice die *in utero* and the embryos have heart abnormalities that include thin and disorganized trabeculae (Varfolomeev et al., 1998), phenotypes also observed upon *TAZ* depletion *in utero* (Phoon et al., 2012). Thus, defects in caspase-8 activity may contribute to the cardiomyopathy in BTHS. The relative capacity of BTHS mitochondria to be consumed by mitophagy has not been reported. However, BTHS lymphocytes have more mitochondria that are individually less functional (Gonzalvez et al., 2013) suggesting that mitochondrial homeostasis may indeed be perturbed in BTHS and contribute to disease progression.

Importantly, the detailed biochemical and cell biologic characterization of the numerous BTHS models have begun to identify potential avenues for therapeutic intervention. For instance, suppression of mitochondrial ROS attenuates the energetic and functional decline caused by *TAZ*-depletion in rodent cardiac myocytes (He et al., 2014) and corrects the sarcomere organization and contractile function of induced BTHS cardiomyocytes (Wang et al., 2014a). Genetic and/or pharmacologic targeting of the lipase that initiates CL remodeling in yeast, flies, and patient lymphoblasts prevents, to varying degrees, the mitochondrial dysfunction caused by the absence of TAZ and can additionally rescue the sterility of male* taz^-/-^* flies (Malhotra et al., 2009a; Baile et al., 2014b; Ye et al., 2014a). These results suggest that the mitochondrial dysfunction stemming from TAZ deficiency is not likely due to reduced remodeled CL, but instead caused by the increased abundance of MLCL and/or the low total amounts of CL. As such, drugs that can scavenge mitochondrial ROS or prevent the accumulation of MLCL (inhibit the activity of the upstream deacylase(s); enhance the activity of other putative MLCL remodelers; augment a pathway that degrades MLCL) could be used to the potential therapeutic benefit of BTHS patients.

### DNAJC19 MUTATIONS LEADING TO DILATED CARDIOMYOPATHY WITH ATAXIA (DCMA) SYNDROME

To date, only two mutations have been identified in *DNAJC19* (DnaJ/Hsp40 homolog, subfamily C, member 19) that are associated with dilated cardiomyopathy with ataxia (DCMA; Davey et al., 2006; Ojala et al., 2012), an autosomal-recessive disorder that presents with early onset dilated cardiomyopathy, non-progressive cerebellar ataxia leading to motor delays, testicular dysgenesis, growth failure, and elevated levels of 3-MGA. Additional features include microcytic anemia, mild to borderline non-progressive mental retardation, hepatic steatosis, and occasional optic atrophy (Davey et al., 2006; Sparkes et al., 2007; Ojala et al., 2012). DCMA shares with BTHS certain clinical features including left ventricular non-compaction with spongey and trabeculated myocardium (Sparkes et al., 2007). However, unlike in BTHS (Barth et al., 2004), DCMA patients do not exhibit neutropenia or skeletal myopathy.

*DNAJC19* is on chromosome 3q26.33 (Davey et al., 2006). The gene consists of six exons and is ubiquitously expressed as a 525 bp transcript with a minor 435 bp form, lacking exon 4, in all tissues tested and control fibroblasts (Davey et al., 2006). The consanguineous Canadian Dariusleut Hutterite families all have an exon4 splicing defect while two Finnish brothers have a frameshift mutation resulting in a truncated protein (Davey et al., 2006; Ojala et al., 2012). Consistent with these mutations, only the shorter transcript is expressed in fibroblasts from one Hutterite patient (Davey et al., 2006) and DNAJC19 protein is not detected in fibroblasts derived from the Finnish siblings (Ojala et al., 2012). Since there are only two genetic variants of *DNAJC19* linked to DCMA, it is not possible to describe genotype–phenotype correlations in this disorder. Still, in a retrospective study of the Hutterite patients that all share the same mutation (Sparkes et al., 2007), 13 of the 17 patients developed dilated cardiomyopathy and 10 later died. Interestingly, three patients had resolved or stabilized cardiomyopathy and another four did not present with cardiac defects at all. Thus, there is significant clinical variability with respect to this particular DCMA allele.

DNAJC19 is associated with the matrix-facing leaflet of the IMM via a predicted NH_2_-terminal transmembrane region (Richter-Dennerlein et al., 2014) and contains a conserved DNAJ domain at the COOH terminus, in contrast to other conventional DNAJ-proteins with an NH_2_-terminal J-domain. DNAJ domain-containing proteins typically act as molecular chaperones for Hsp70/Hsp40s and prevent protein aggregation by aiding in the folding and assembly of newly synthesized proteins (Ohtsuka and Hata, 2000; Mayer and Bukau, 2005). Sequence alignment indicates that DNAJC19 is orthologous to yeast Pam18p, a constituent of the TIM23 import machinery (Mokranjac et al., 2003). In yeast, the Pam18p and Mdj2p proteins are essential components of the TIM23 translocation machinery (Mokranjac et al., 2005), interacting with mitochondrial Hsp70p, Pam16p, and Tim44p to form the presequence translocase-associated motor complex (Schneider et al., 1994; Moro et al., 2002; Truscott et al., 2003; Frazier et al., 2004; Kozany et al., 2004; D’Silva et al., 2008). This subcomplex associates with the core TIM23 translocon (consisting of Tim23p, Tim17p, Tim50p and variably, Tim21p) and mediates import of precursors destined for the matrix in an ATP- and membrane potential-dependent manner (Bomer et al., 1997; Moro et al., 1999; Geissler et al., 2002; Yamamoto et al., 2002; Chacinska et al., 2005; van der Laan et al., 2007). Thus, DNAJC19 may, like yeast Pam18p, stimulate the ATPase activity of mtHsp70 and stabilize mtHsp70 binding to incoming peptides. For an excellent review on mitochondrial protein translocation, refer to (Chacinska et al., 2009).

Consistent with a role in protein import, DNAJC19 interacts with MAGMAS, the conserved mammalian ortholog of yeast Pam16p that is essential for development (Gonczy et al., 2000; Jubinsky et al., 2001, 2003; D’Silva et al., 2005). MAGMAS is structurally similar to DNAJC19 but peripherally associated with the matrix-leaflet of the IMM. The interaction between DNAJC19 and MAGMAS occurs via their reciprocal J-domains and is required to recruit DNAJC19 to the core TIM23 translocon (Sinha et al., 2010). MAGMAS then associates with TIM17, a subunit of the TIM23 core, to form the TIM23 translocation machinery (Mehawej et al., 2014; Sinha et al., 2014). Interestingly, there are three distinct forms of TIM23 translocon that incorporate different TIM17 isoforms and associate with either DNAJC15 (TIM17a; translocase A), another co-chaperone of the same Hsp40-type (Hatle et al., 2013; Schusdziarra et al., 2013), or DNAJC19 (TIM17b_1_ and TIM17b_2_; translocase Bs). Of the three versions of TIM23, translocase Bs are critical for basal mitochondrial biogenesis (e.g., OXPHOS, iron–sulfur cluster biogenesis, mtDNA copy number, and maintenance of mitochondrial membrane potential) while translocase A plays a dispensable, albeit supportive role when translocase Bs are absent (Sinha et al., 2014). In light of these results, defects in mitochondrial presequence protein import and consequently, mitochondrial biogenesis, may represent the mechanism of DCMA pathogenesis.

Recently, however, human and murine DNAJC19 were found to additionally interact with prohibitin (PHB complexes) (Richter-Dennerlein et al., 2014). PHB complexes are large hetero-oligomeric complexes composed of PHB1 and PHB2 subunits (Tatsuta et al., 2005) that are involved in cristae morphogenesis (Merkwirth et al., 2008) and modeled to function as lipid scaffolds in the IMM, redistributing lipids such as CL (Christie et al., 2011) and delineating functional membrane domains (Osman et al., 2009b). In yeast, Δ*phb1* is synthetically lethal with Δ*crd1* highlighting the importance of conserved PHB complexes in CL metabolism (Osman et al., 2009a). Interestingly, *DNAJC19*-depletion in HEK293T cells shifts the CL acyl profile to longer and less saturated chains; however, CL levels are unaffected and MLCL does not accumulate (Richter-Dennerlein et al., 2014). Moreover, while a functional DNAJ-domain is not required for DNAJC19 to interact with PHB complexes, it is necessary to rescue the changes in CL molecular composition that occur upon *DNAJC19* knockdown (Richter-Dennerlein et al., 2014). In contrast, *PHB2* knockdown results in the same three biochemical alterations that characterize BTHS (increased MLCL, decreased CL, and changes in CL acyl chain composition), although the increase in MLCL is significantly less than in *TAZ* knockdown cells (Richter-Dennerlein et al., 2014). Concomitant knockdown of *PHB2* or *DNAJC19* with* TAZ* does not alter the accumulation of MLCL that occurs upon depletion of* TAZ* alone, indicating that neither PHB complexes nor DNAJC19 are required to generate the substrate, MLCL, used by TAZ. Combined, these results suggest unexpected roles for both PHB complexes and DNAJC19 in CL remodeling and further indicate that their functions in this regard, are at least partially distinct. TAZ does not interact with PHB2 or DNAJC19 directly (Richter-Dennerlein et al., 2014). Thus, the ability of PHB/DNAJC19 complexes to define specific membrane domains, such as those with negative curvature, is postulated to confer acyl chain specificity to TAZ (Schlame et al., 2012a; Richter-Dennerlein et al., 2014). In the absence of such privileged domains, TAZ remodeling still occurs, it just lacks acyl chain specificity.

**FIGURE 5:**
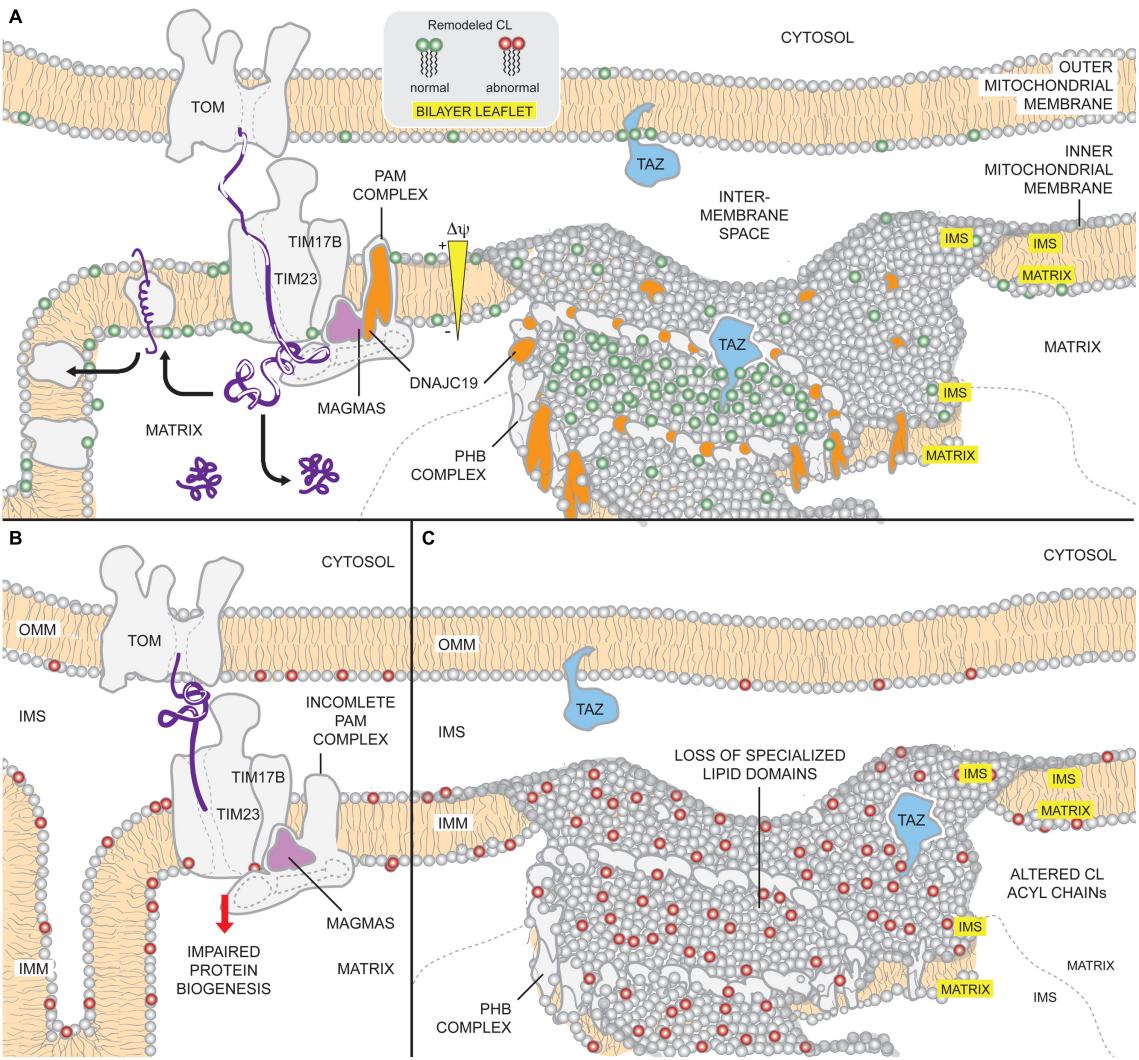
**Potential mechanisms of DCMA mitochondrial dysfunction. (A)** Under physiological conditions, DNAJC19 is targeted to TIM17B by MAGMAS and associates with components of the TIM23 translocation machinery, forming translocase B. DNAJC19 is homologous to yeast Pam18p which stimulates the mtHsp70 activity of the PAM (presequence-associated motor) complex and stabilizes binding of incoming precursors. Moreover, DNAJC19 also interacts with prohibitin-2 (PHB) of the PHB complexes. PHB1/PHB2 oligomers form ring-like complexes that are modeled to delineate specialized membrane domains. Functional segregation of CL and TAZ in such domains may confer acyl chain specificity to TAZ, allowing it to perform physiologically relevant CL remodeling (green). Thus, DNAJC19 may participate in both mitochondrial presequence protein import as well as formation of membrane domains that are important for TAZ-based CL remodeling. **(B)** In the absence of DNAJC19, the ability of the TIM23 machinery to import proteins across the IMM may be compromised. Consequently, the biogenesis of mitochondrial proteins, such as subunits of respiratory complexes, may be reduced. **(C)** Further, loss of DNAJC19 prevents the PHB complex-based generation of privileged membrane domains. In the absence of such domains, TAZ remodeling, which may still occur, lacks specificity (red). For clarity, not all components of the TOM, TIM, and PAM complexes are depicted.

In sum, it appears that DNAJC19 has dual functions in the regulation of CL remodeling and mitochondrial protein biogenesis (**Figure 5**). A key question that remains unresolved is whether both activities contribute to DCMA disease pathogenesis. Presently lacking is any information regarding the lipid profile and protein import functionality of mitochondria isolated from actual DCMA patients; such data is needed to better understand the underlying pathogenic mechanism. If similar alterations in CL are detected in DCMA patient cells, future studies will be needed to define how DNAJC19/PHB complexes regulate the collection of acyl chains attached to CL and relate these changes to mitochondrial dysfunction. This latter question is all the more interesting and relevant given that in yeast, flies, and mammalian cells, genetically and/or pharmacologically preventing production of MLCL by targeting the lipase that begins the remodeling cascade rescues the multitude of phenotypes attributed to TAZ deficiency (Malhotra et al., 2009a; Baile et al., 2014b; Ye et al., 2014a).

### SERAC1 MUTATIONS LEADING TO MEGDEL SYNDROME

Evidence that PG, like CL, undergoes physiologically important remodeling was recently provided by the identification of *serine active site containing 1* (*SERAC1*) mutations that cause autosomal-recessive MEGDEL syndrome [3-MGA, sensorineural deafness, encephalopathy, and neuroradiological evidence of progressive Leigh-like syndrome (Wortmann et al., 2006, 2012)]. In addition to classical MEGDEL symptoms, an increasing list of clinical phenotypes have been associated with *SERAC1* mutations; infantile mitochondrial hepatopathy, psychomotor and developmental delay, bilateral optic nerve atrophy, myoclonic epilepsy, and microcephaly (Sarig et al., 2013; Lumish et al., 2014; Vilarinho et al., 2014; Wedatilake et al., 2014). Similar to BTHS and DCMA, MEGDEL patients have 3-MGA and variable mitochondrial dysfunction.

*SERAC1* is located on chromosome 6q25.3 and the encoded protein resides in the MAM (Wortmann et al., 2012). SERAC1 is a predicted single-pass transmembrane protein that is 654 amino acids long and translated from 17 exons into three isoforms (Wortmann et al., 2012). The protein is a member of the PGAP (post-GPI attachment to protein 1)-like protein domain family and contains an α/β-hydrolase fold and a highly conserved serine-lipase domain (Wortmann et al., 2012). To date, 18 different mutations have been described in 24 patients, many of which are frameshift, nonsense, or missense mutations within or upstream of the lipase domain (Karkucinska-Wieckowska et al., 2011; Wortmann et al., 2012; Sarig et al., 2013; Tort et al., 2013; Dweikat et al., 2014; Lumish et al., 2014; Vilarinho et al., 2014; Wedatilake et al., 2014). Of note, a patient recently described with severe, early onset MEGDEL symptoms harbors compound frameshift and stop-gain heterozygous mutations upstream of the lipase domain (Lumish et al., 2014). In contrast to patients with variants within the lipase domain, which may allow for a protein with residual activity, the production of truncated SERAC1 completely lacking the lipase domain from both alleles may account for the severity of the particular patient’s phenotype.

SERAC1 is implicated in changing the acyl chain composition of CL’s precursor, PG. Specifically, MEGDEL patient fibroblasts have elevated concentrations of PG-34:1 and lower concentrations of PG-36:1; the acyl chain compositions of the other major phospholipid classes, with the notable exception of CL, are normal (Wortmann et al., 2012). The inability to convert PG-34:1 to PG-36:1 results in the accumulation of PG-34:1 and subsequent incorporation of PG with these acyl chain species into CL. Thus, in MEGDEL patients, CL levels are normal but the acyl chain composition of CL is altered (Wortmann et al., 2012). As in the case of DNAJC19 deficiency, if and how normal levels of CL of abnormal acyl chain composition affects mitochondrial function is at present unclear. Notably, most MEGDEL patient tissues and fibroblasts exhibit OXPHOS dysfunction (Wortmann et al., 2006, 2012; Karkucinska-Wieckowska et al., 2011; Sarig et al., 2013; Tort et al., 2013; Dweikat et al., 2014; Wedatilake et al., 2014), though the degree of impairment and the affected respiratory chain component(s) varies. Moreover, ROS production is increased and catalase levels decreased in fibroblasts derived from at least one MEGDEL patient (Karkucinska-Wieckowska et al., 2011); therefore, similar to BTHS, defective OXPHOS function may cause an imbalance in redox homeostasis in MEGDEL patients. However, additional basic work is required to define whether increased ROS production and reduced scavenging is a consistent feature associated with SERAC1 dysfunction, exactly how defective SERAC1 variably impairs OXPHOS, and if the changes noted in the acyl chain composition of CL in MEGDEL patients are detrimental to mitochondrial functionality.

It is curious to note that endogenous TAZ, which is presumably functional in MEGDEL patients, is unable to correct the unusual CL acyl chains that accumulate in the absence of SERAC1 function. PG is a precursor of CL and therefore contributes directly to the collection of acyl chains associated with pre-remodeled CL. Since other phospholipids have normal acyl chain compositions in MEGDEL patients (Wortmann et al., 2012), in theory, TAZ should still be able to generate “normal” CL. One possible explanation for this discrepancy is that perhaps the CL that accumulates in MEGDEL patients is not a substrate for the lipase that functions upstream of TAZ to initiate CL remodeling.

**FIGURE 6:**
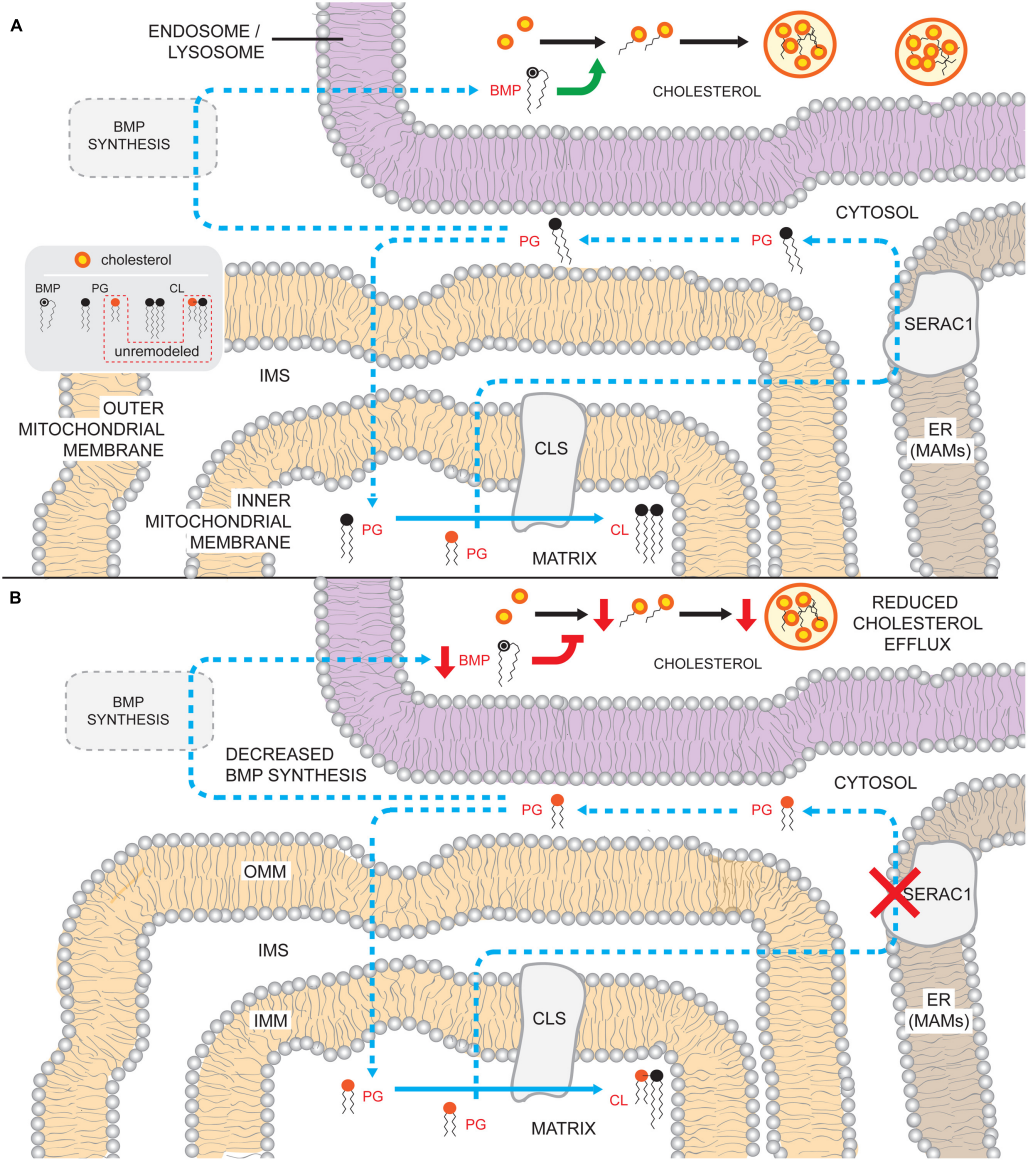
**Potential consequences of the absence of SERAC1 activity. (A)** PG that is generated on the matrix-leaflet of the IMM is trafficked out of the mitochondrion and remodeled by SERAC1 on the ER MAMs. Remodeled PG can subsequently serve as substrate for BMP and CL synthesis. BMP in the endosomsal/lysosomal compartments regulates (green arrow) cholesterol esterification and trafficking out of the compartment. **(B)** In the absence of a functional SERAC1, PG is not remodeled and accumulates shorter acyl chain moieties. The decrease in acyl chain length somehow reduces overall BMP levels, perhaps because the unremodeled PG is a poor precursor for BMP synthesis. Reduced BMP levels in the endosome/lysosome impairs cholesterol esterification (inhibition in red) and leads to decreased (red arrows) cholesterol eﬄux. The lack of SERAC1 also changes the steady state composition of CL acyl chains. Blue arrows denote enzymatic reactions and lipid movements. Dashed arrows describe uncharacterized steps and pathways.

While SERAC1 defects do not affect the abundance or acyl chain pattern of phosphatidylcholine, phosphatidylserine, or PE, it does significantly reduce levels of BMP (Wortmann et al., 2012), a lipid implicated in endosomal/lysosomal homeostasis and function (Hullin-Matsuda et al., 2009; **Figure 6**). BMP levels regulate cholesterol trafficking with low intracellular BMP causing accumulation of free cholesterol in late endosomes. Indeed, SERAC1 patient fibroblasts accumulate unesterified cholesterol (Sarig et al., 2013; Tort et al., 2013) and patient biopsies show dramatically disorganized striated muscle ultrastructure with abnormal mitochondria and lysosomal accumulation of neutral fat droplets (Wortmann et al., 2012; Wedatilake et al., 2014). It is presently not clear how a change in the acyl chain composition of PG translates into lower amounts of BMP especially since the molecular composition of BMP in MEGDEL patients is not altered relative to controls (Wortmann et al., 2012). Also unclear is how PG produced on the matrix side of the IMM gains access to SERAC1, which resides in the MAM. As our understanding of how SERAC1 regulates the acyl chain composition of CL and the production of BMP is in its infancy, future basic work is needed to develop assorted models designed to better understand these processes and how their disturbance cause the numerous MEGDEL syndrome phenotypes.

### ACYLGLYCEROL KINASE MUTATIONS LEADING TO SENGERS SYNDROME

Sengers syndrome is caused by the absence of the IMS-residing AGK (Calvo et al., 2012; Mayr et al., 2012; Haghighi et al., 2014). Symptoms of Sengers syndrome may present at birth, childhood, or early adulthood and its clinical manifestations range from being severe, causing death in infancy, to mild, allowing survival into adulthood (van Ekeren et al., 1993; Mayr et al., 2012). This autosomal-recessive disorder is characterized by congenital cataracts, hypertrophic cardiomyopathy, skeletal myopathy, exercise intolerance, lactic acidosis, and increased urinary 3-MGA; though motor development is delayed, mental development of the affected individuals are normal (Sengers et al., 1975). Hence, the phenotypic spectrum associated with Sengers syndrome substantially overlaps with both Barth and DCMA syndromes. Sengers syndrome was originally thought to be due to deficiencies in the level and/or function of adenine nucleotide translocase 1 (ANT1; Jordens et al., 2002). ANT1 is the heart/muscle isoform of the mitochondrial ADP/ATP exchanger that plays a fundamental role in OXPHOS by mediating the flux of ADP and ATP across the IMM (Li et al., 1989). However, genetic analyses excluded mutations in the *ANT1* gene; as such, it was subsequently proposed that transcriptional, translational, or post-translational events might be responsible for the lower amounts of ANT1 observed in patients (Jordens et al., 2002). Recently, two groups localized the defect *via* exome sequencing, not in or around *ANT1*, but instead to the *AGK* locus on chromosome 7q34 (Calvo et al., 2012; Mayr et al., 2012).

The *AGK* gene contains 17 exons and encodes for a 422 amino acid protein; it is a mitochondrial membrane-associated, multi-substrate lipid kinase that contains domains that are highly homologous to sphingosine kinase-2 as well as DAG kinase (Topham and Prescott, 1999; Waggoner et al., 2004; Bektas et al., 2005). When phylogenetic analyses of AGK were undertaken, sequence comparisons to other lipid kinases such as DAG, ceramide, and sphingosine kinases revealed that despite containing a highly conserved DAG kinase catalytic domain, human and murine AGK segregate to a unique branch and are not members of any previously described lipid kinase family (Waggoner et al., 2004).

Like Barth and DCMA syndromes, genotype–phenotype correlations in Sengers syndrome are not readily apparent. For instance, there are Sengers syndrome patients who are non-syndromic or do not develop lactic acidosis (Aldahmesh et al., 2012), and siblings who share the same mutation but present with very different disease courses [one had the severe form of disease and died at 15 months of age while the other brother, at 3 years old, was without skeletal myopathy or physical limitations (Siriwardena et al., 2013)]. Thus, the etiology of Sengers syndrome is also likely to involve genetic modifiers that can significantly affect disease progression and severity.

However, there is evidence that a genotype–phenotype correlation for Sengers syndrome patients potentially exists (Haghighi et al., 2014). Two forms of the syndrome have been described, a severe neonatal form that leads to infantile death (Smeitink et al., 1989) and a more benign, but chronic, form that has allowed survival into the fourth decade (van Ekeren et al., 1993). The former disease type is always associated with homozygous *AGK* nonsense mutations while in the latter, patients usually harbor at least one splice site variant or a start codon mutation, but not doubly null/missense alleles (Haghighi et al., 2014). Presumably, aberrant splicing of the COOH terminal exons (in or after the DAG kinase domain) produces proteins with residual AGK activity while inactivation of the canonical start site allows for initiation at an alternative/cryptic start site (Starck et al., 2012). In support of this possible genotype–phenotype relation, the oldest surviving patients (>35 years) carry either heterozygous start site and stop codon mutations or a homozygous mutation affecting exon 15–16 splicing (Lalive D’Epinay et al., 1986; van Ekeren et al., 1993).

A role for AGK in the assembly, stability and/or regulation of OXPHOS components such as ANT1 and complex I has been speculated (Pitkanen et al., 1996; Haghighi et al., 2014). OXPHOS defects in patient samples have been variable (Pitkanen et al., 1996; Jordens et al., 2002; Morava et al., 2004; Aldahmesh et al., 2012; Calvo et al., 2012; Mayr et al., 2012; Siriwardena et al., 2013; Haghighi et al., 2014). In the more severe cases, ANT1 and complex I levels are low and the mtDNA copy number reduced. However, in the milder cases, OXPHOS function is spared. Curiously, ANT1 levels are normal in undifferentiated myoblasts but are dramatically reduced upon myoblast differentiation (Mayr et al., 2012). This observation is notable as it suggests that ANT1 stability is compromised during muscle fiber differentiation in Sengers syndrome patients. While the exact molecular function of AGK is not known, its potential role in CL metabolism is consistent with the varied bioenergetic impairments, structurally abnormal mitochondria, and lipid and glycogen deposits in patient-derived skeletal and cardiac muscle (Sengers et al., 1975; Siriwardena et al., 2013). A comprehensive phospholipid analysis has not been performed for Sengers syndrome cells and/or tissues. Therefore, whether the absence of AGK function correlates with disturbances in phospholipid metabolism has not yet been established. If AGK generates PA that contributes substantially to TAMM41’s substrate pool (**Figure 1**), then the absence of AGK function should significantly impact downstream PG and CL synthesis. Given the aforementioned results concerning the relative stability of ANT1 in undifferentiated *versus* differentiated myoblasts, the choice of cell/tissue to analyze is likely to be critically important in uncovering if and how AGK participates in mitochondrial phospholipid metabolism. Such information is anticipated to shed crucial light onto Sengers syndrome disease pathogenesis.

### PHOSPHATIDIC ACID-PREFERRING PHOSPHOLIPASE A_1_ MUTATIONS LEADING TO HEREDITARY SPASTIC PARAPLEGIA

Mutations in the phospholipase A_1_ family members *DDHD1* (Bouslam et al., 2005; Tesson et al., 2012; Liguori et al., 2014) and *DDHD2* (Schuurs-Hoeijmakers et al., 2012; Gonzalez et al., 2013; Citterio et al., 2014; Doi et al., 2014; Magariello et al., 2014) have recently been linked to autosomal-recessive forms of hereditary spastic paraplegia (HSP). HSPs are an extremely heterogeneous group of inherited neurological disorders that are classified by patients who present with (complex HSP) or without (uncomplicated or pure HSP) neurological defects. Pure HSP is characterized by progressive spasticity and weakness of the lower limbs. In complex HSP, spastic paraplegia is compounded with neurological and systemic abnormalities such as dementia, intellectual disability, ataxia, and neuropathy, amongst others [see (Fink, 2013) for comprehensive discussion of HSP and spastic paraplegia variants]. Furthermore, there is varied disease onset, progression, and “functional plateaus” (a point at which there is very little further disability) between the HSPs and as such, there is very little genotype–phenotype correlation in this heterogeneous disorder.

Hereditary spastic paraplegia has been linked to over 50 genetic loci, covering all chromosomes, of which only 22 genes have been identified so far (Finsterer et al., 2012; Fink, 2013). Two of the identified disease-associated genes are *DDHD1* and *DDHD2* that encode protein products with PA-phospholipase A_1_ activity. *DDHD1* is localized to chromosome 14q21.1, contains 12 exons (Tesson et al., 2012) and encodes for the 872 amino acid protein, PA-PLA_1_. Initial characterization identified substantial PA-PLA_1_ activity in bovine brain and testis that corresponds to the two tissues with the highest *DDHD1* mRNA expression (Higgs and Glomset, 1994; Higgs et al., 1998). In humans, these two tissues, along with heart and skeletal muscle, additionally contain high levels of *DDHD2*, a more ubiquitously expressed DDHD family member (Nakajima et al., 2002; Morikawa et al., 2009; Sato et al., 2010). DDHD2 is both cytosolic and membrane-associated (Sato et al., 2010; Baba et al., 2013). Located on chromosome 8p11.23 and containing 18 exons, *DDHD2* is expressed as two major mRNA transcripts that correspond to the full length 711 amino acid protein and another that lacks the COOH terminal DDHD phospholipase domain. When patient *ddhd2* mutants were overexpressed in and partially purified from HEK293T cells, PA-PLA_1_ activity was either low or absent compared to overexpressed wt DDHD2 (Doi et al., 2014). Notably, depletion of PA-PLA_1_ activity (DDHD1 or DDHD2) in some, but not all cells, can cause mitochondrial elongation (Schuurs-Hoeijmakers et al., 2012; Baba et al., 2013, 2014), presumably due to unopposed fusion. Thus, one might expect that the balance between fission and fusion may be disturbed in some cells in patients with *ddhd1* or *ddhd2* mutations with an overall shift toward too much fusion. As active fission is a crucial process for clearance of damaged mitochondria by mitophagy, unopposed fusion could drive the accumulation of dysfunctional mitochondria (Twig et al., 2008). Consistent with the possible accretion of sub-optimal mitochondria, respiration and ATP levels are decreased and cytosolic ROS increased in *ddhd1* patient-derived lymphoblasts (Tesson et al., 2012). Unfortunately, PA levels and mitochondrial morphology have not been documented in either *ddhd1* or *ddhd2* patient-derived cells (Tesson et al., 2012) but there are no major changes in phospholipids in *ddhd2^-/-^* mouse brains (Inloes et al., 2014).

It is unclear whether or not depletion of DDHD1 or DDHD2 can directly affect mitochondrial dynamics by accumulating pro-fusogenic PA on the OMM. Given their similar substrate specificity, they may have redundant roles in this regard. Still, there might be functional differences for these two PA phospholipases that are dictated by their expression patterns and subcellular distributions. As such, future studies are needed to investigate the potential causative link between dysregulation of PA metabolism on the OMM with disease pathogenesis for these subtypes of HSP.

## PERSPECTIVES

Phospholipids are not simply cellular barriers that delineate cells and organelles thus allowing biochemical pathways to be compartmentalized and cross-regulated. Although predominantly synthesized in the ER, some phospholipids, such as PG and CL, are exclusively made in the mitochondrion, while others, such as PE, involve a pathway that begins in the ER but is completed in the cell’s powerhouse. In mitochondria, phospholipids are important for an impressive array of functions. Indeed, when phospholipid levels, molecular species, or distribution within the mitochondrion is affected, as is the case for the diseases discussed herein, mitochondrial dysfunction ensues. BTHS was the first known inborn error of mitochondrial phospholipid metabolism. Since the identification of *TAZ* as the causative gene for BTHS, exome sequencing and basic research have both contributed to the recent addition of new diseases that impinge upon mitochondrial lipid metabolism. We propose to classify these disorders as a new category of mitochondrial disease that specifically impacts phospholipid homeostasis. The clinical heterogeneity of patient phenotypes reflects the variety of functions that phospholipids partake in and their fundamental importance for mitochondrial physiology. Future work needs to focus on developing appropriate models for these diseases. As exemplified by BTHS, the availability and in-depth characterization of such models will contribute enormously to our understanding of each disease process and identify potential therapeutic strategies. Further, such basic work should help us better understand disease mechanisms and provide explanations for shared and distinct phenotypes.

## Conflict of Interest Statement

The authors declare that the research was conducted in the absence of any commercial or financial relationships that could be construed as a potential conflict of interest.
